# Pradimicin U*,* a promising antimicrobial agent isolated from a newly found *Nonomuraea composti* sp. nov

**DOI:** 10.1038/s41598-024-60744-w

**Published:** 2024-05-13

**Authors:** Thitikorn Duangupama, Pattama Pittayakhajonwut, Chakapong Intaraudom, Chanwit Suriyachadkun, Sarin Tadtong, Nattakorn Kuncharoen, Ya-Wen He, Somboon Tanasupawat, Chitti Thawai

**Affiliations:** 1 Department of Biology, School of Science, King Mongkut’s Institute of Technology Ladkrabang, Bangkok, 10520 Thailand; 2 National Center for Genetic Engineering and Biotechnology (BIOTEC), National Science and Technology Development Agency (NSTDA), 113 Thailand Science Park, Phaholyothin Road, Khlong Nueng, Khlong Luang, 12120 Pathum Thani Thailand; 3 Thailand Bioresource Research Center (TBRC), National Center for Genetic Engineering and Biotechnology (BIOTEC), National Science and Technology Development Agency (NSTDA), 113 Thailand Science Park, Phaholyothin Road, Khlong Nueng, Khlong Luang, 12120 Pathum Thani Thailand; 4 Department of Pharmacognosy, Faculty of Pharmacy, Srinakharinwirot University, Nakhon Nayok, 26120 Thailand; 5 Department of Plant Pathology, Faculty of Agriculture, Kasetsart University, Bangkok, 10900 Thailand; 6 State Key Laboratory of Microbial Metabolism, School of Life Sciences and Biotechnology, Shanghai Jiao Tong University, Shanghai, 200240 People’s Republic of China; 7 Department of Biochemistry and Microbiology, Faculty of Pharmaceutical Sciences, Chulalongkorn University, Bangkok, 10330 Thailand; 8 Actinobacterial Research Unit, School of Science, King Mongkut’s Institute of Technology Ladkrabang, Bangkok, 10520 Thailand

**Keywords:** *Nonomuraea*, Polyphasic taxonomic characterization, Whole-genome sequence analysis, Pradimicin U, Biotechnology, Drug discovery, Microbiology

## Abstract

Pradimicin U is a new dihydrobenzo[a]naphthacenequinone compound found to be active on a screen designed to investigate compounds with antimicrobial activity, produced by the actinomycete designated strain FMUSA5-5^T^. The strain was isolated from a bio-fertilizer of *Musa* spp. collected from Suphanburi province, Thailand. The chemotaxonomic characteristics and 16S rRNA gene analysis revealed that strain FMUSA5-5^T^ is a member of the genus *Nonomuraea.* Low genome-based taxonomic criteria, average nucleotide identity (ANI) (82.8–88.3%), average amino*-*acid identity (AAI) (79.4–87.3%)*,* and digital DNA–DNA hybridization (dDDH) (29.5–38.5%) values and several phenotypic differences between strain FMUSA5-5^T^ and its closest type strains of the genus *Nonomuraea* indicated that strain FMUSA5-5^T^ represents a novel species of the genus *Nonomuraea* and the name *Nonomuraea composti* sp. nov. is proposed for the strain. The crude extract from the culture broth of strain FMUSA5-5^T^ displayed promising antimicrobial activity against several pathogens and led to the isolation of a novel secondary metabolite, pradimicin U. Interestingly, this compound displayed a broad spectrum of biological activities such as antimalarial activity against *Plasmodium falciparum* K1 (IC_50_ value = 3.65 µg/mL), anti-*Mycobacterium tuberculosis* H37Ra (MIC value = 25.0 µg/mL), anti-*Alternaria brassicicola* BCC 42724 (MIC value = 25.0 µg/mL), anti-*Bacillus cereus* ATCC 11778 and anti-*Staphylococcus aureus* ATCC 29213 (MIC values = 6.25 and 1.56 µg/mL, respectively). Moreover, the compound possessed strong anti-human small cell lung cancer (NCI-H187) activity with IC_50_ value of 5.69 µg/mL, while cytotoxicity against human breast cancer (MCF-7) and Vero cells was very weak (IC_50_ values of 52.49 and 21.84 µg/mL, respectively).

## Introduction

The genus *Nonomuraea* is an important genus of actinobacteria that belongs to the order *Streptosporangiales* and the family *Streptosporangiaceae* within the class *Actinomycetia*^1,2^. Typically, *Nonomuraea* species produce branched substrate and aerial mycelia, and a variety of spore chains, hooked, spiral, or straight, could be found directly on the aerial mycelium. *Meso*-diaminopimelic acid (DAP) and madurose are generally detected in cell hydrolysates. Diphosphatidylglycerol, phosphatidylethanolamine, sugar-containing phospholipid, and ninhydrin-positive phospholipid are found to be contained in cell membranes. A series of menaquinones, MK-9 (H_4_), MK-9 (H_2_), and MK-9 (H_0_), are usually detected in this genus^3^. *Nonomuraea* species are primarily known for their production of bioactive compounds, particularly antibiotics, and have been the subject of research in biotechnology and pharmaceutical sciences. To date, with continual isolation and identification, many bioactive secondary metabolites have been identified from *Nonomuraea* spp. For example, nonocarboline from *Nonomuraea* sp. 1808210CR could inhibit the growth of *Bacillus subtilis* with a MIC value of 4.2 µg/mL^4^. Phthalic acid, a promising natural product from *Nonomuraea* sp. VAS16, possessed antibacterial activity against *Yersinia enterocolitica* and *Vibrio parahaemolyticus* (31.25 µg/mL)^5^. Brartemicin, a trehalose-derived metabolite from *Nonomuraea* sp. TP-A0870, was reported as a strong cytotoxic agent against murine colon carcinoma 26-L5 cells with IC_50_ = 0.39 µM^6^. Thus, the isolation of *Nonomuraea* species in nature can lead to the discovery of bioactive compounds with therapeutic applications. In our continuing search for novel secondary metabolite-producing actinomycetes from natural sources, a *Nonomuraea*-like strain, designated strain FMUSA5-5^T^, was isolated from bio-fertilizer made from *Musa* spp. This strain was taxonomically characterized using a polyphasic approach. The biosynthetic gene clusters (BGCs) were also predicted from the genome of strain FMUSA5-5^T^. Furthermore, we found that the crude extract of strain FMUSA5-5^T^ displayed a broad spectrum of biological activities, such as antimalarial activity against *Plasmodium falciparum* strain K1 (IC_50_ value = 31.25 µg/mL), anti-TB activity against *Mycobacterium tuberculosis* H37Ra (MIC value = 62.5 µg/mL), antibacterial activity against *Bacillus cereus* ATCC 11778 and *Staphylococcus aureus* ATCC 29213 (MIC values = 62.5 and 31.25 µg/mL, respectively), and antioxidant activity (IC_50_ value = 125 µg/mL). Hence, the chemical investigation of the crude extract from the fermentation broth of strain FMUSA5-^T^ was performed. The chemical study found a new compound, namely pradimicin U, as a major component in the culture broth of strain FMUSA5-5^T^. Additionally, biological activities of a pure compound, including antioxidant, antifungal, and cytotoxicity against cancerous (MCF-7 and NCI–H187) and non-cancerous (Vero) cell lines of the isolated compound, were also performed.

## Results and discussion

### Polyphasic taxonomic characterization of strain FMUSA5-5^T^

**Figure 1:**
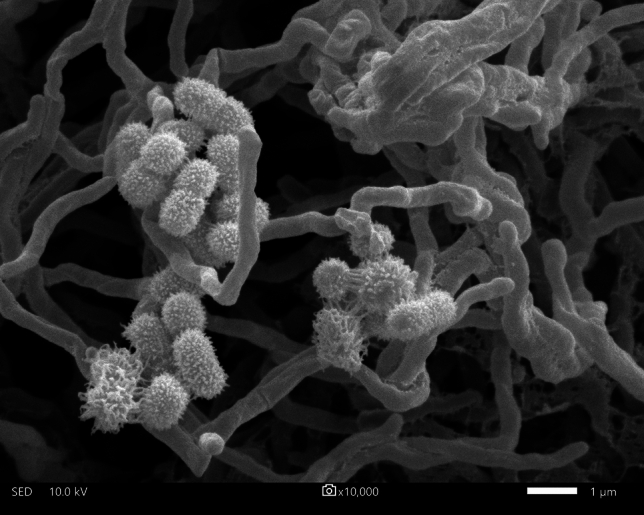
Scanning electron micrograph of strain FMUSA5-5^T^ grown on ISP 2 agar for 30 days at 30 °C. Bar, 1 μm.

**Figure 2:**
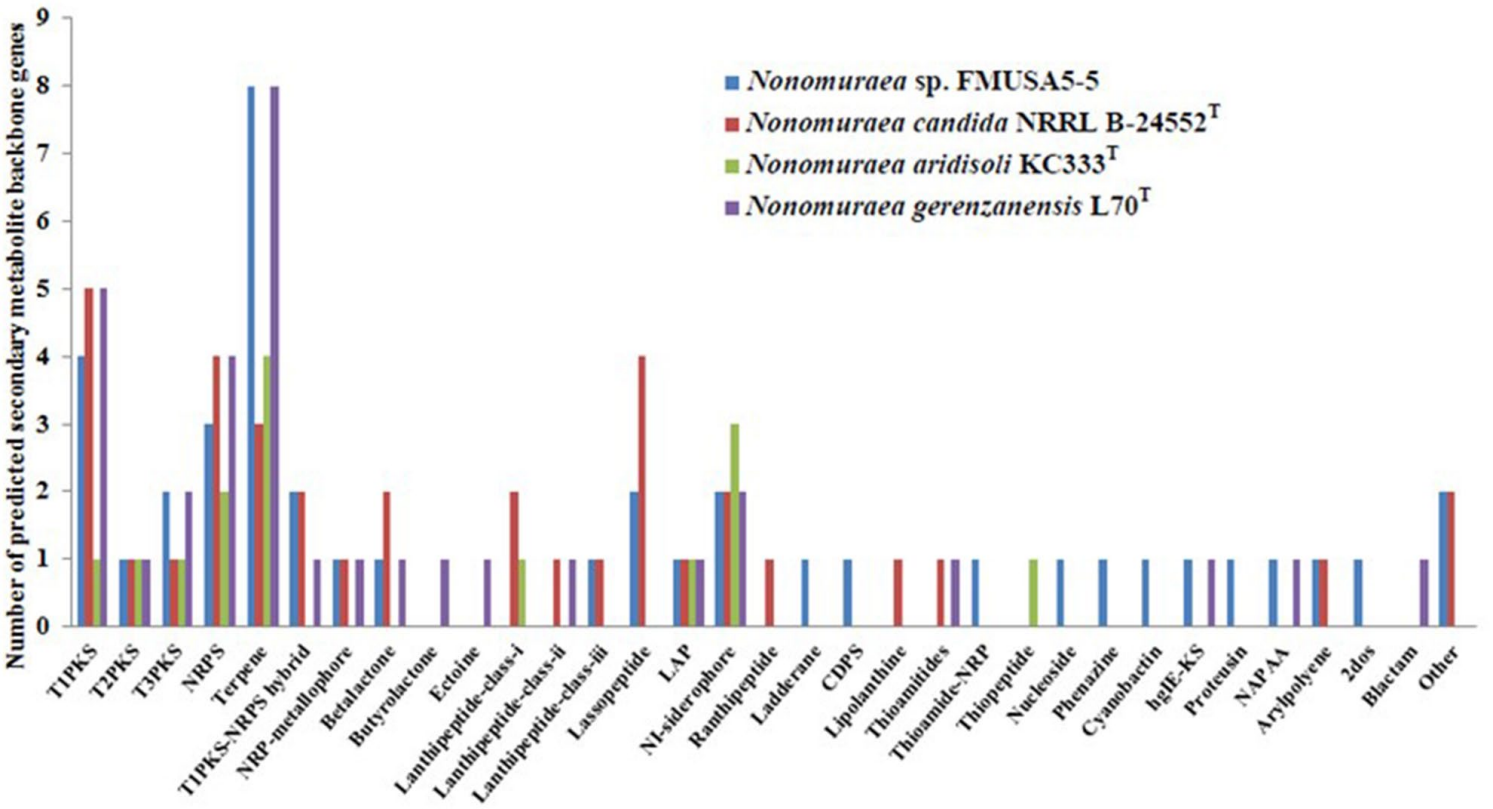
Biosynthetic gene clusters presented in strain FMUSA5-5^T^ and its closest type strains using antiSMASH 7.0.

**Figure 3:**
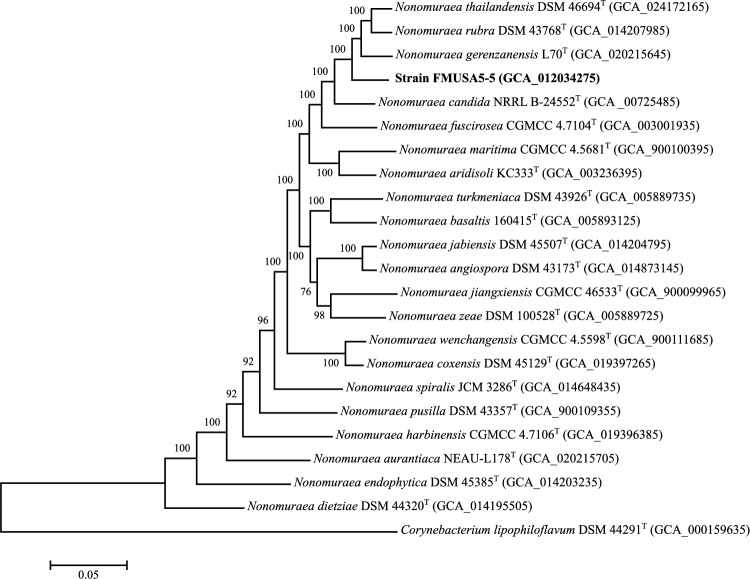
Phylogenomic analysis of strain FMUSA5-5^T^ and type strains affiliated to the genus *Nonomuraea* based on 100 bacterial conserved single copied gene sets of the members. The bootstrap values on the nodes are displayed by > 50.

Bio-fertilizers are natural substances that contain living microorganisms, which applied to the soil or plant, enhance its fertility and promote plant growth. The microorganisms surviving in bio-fertilizers can improve nutrient uptake by plants, solubilize phosphorus, and are considered as a source of many bioactive secondary metabolites against several plant pathogens^7^. During the production process for bio-fertilizers, a high temperature (⁓60–75 °C) and low oxygen content were detected in the compost pile. This condition is not conducive to the survival of aerobic microorganisms. Thus, actinomycetes living in the bio-fertilizers are expected to be different from other actinomycetes. Strain FMUSA5-5^T^ was isolated from a bio-fertilizer of *Musa* spp. collected from Suphanburi province, Thailand. The strain could grow well on ISP 2, ISP 3, and nutrient media. Moderate growth was detected on ISP 4, ISP 5, ISP 7, and Czapek’s sucrose. The growth was poor on ISP 6. The color of substrate mycelium was dark reddish-brown on ISP 2 medium. Aerial mycelium was hardly observed on any media tested for 14 days, but white aerial mycelium could be observed on ISP 2 after 30 days of cultivation at 30 °C. Light brown to reddish brown diffusible pigments were detected on ISP 2, ISP 3, ISP 4, ISP 5, ISP 6, Czapek’s sucrose and nutrient media (Table S1). Strain FMUSA5-5^T^ produced a cluster of rod-shaped spores (0.8–1.0 × 1.0–2.0 µm) with hairy surfaces borne directly on aerial mycelia (Fig. 1). Whole-cell hydrolysates of strain FMUSA5-5^T^ contained *meso*-diaminopimelic acid along with ribose, glucose, mannose, and madurose as the cell-wall sugars. It is known that madurose (3-*O*-methyl-D-galactose) is the diagnostic sugar found in *Nonomuraea* species^3^. Phosphatidylethanolamine (PE), phosphatidylmethylethanolamine (PME), phosphatidylglycerol (PG), phosphatidylinositol mannoside (PIM), three phosphoglycolipids (PGLs), and five unidentified phospholipids (PLs) are the phospholipids which match quite well with those reported by Minnikin et al.^8^ for the genus *Nonomuraea* (Fig. S1). MK-9(H_4_) (70.6%) and MK-9(H_2_) (16.5%) were the major menaquinones while MK-9(H_0_) (8.6%) and MK-9(H_6_) (4.3%) were detected as minor components. Whole-cell fatty acid analysis revealed strain FMUSA5-5^T^ contained primarily *iso*-C_16:0_ (19.4%), 10-methyl C_17:0_ (15.8%), C_16:0_ (10.2%), C_17:0_ (7.1%) (Table S2). The fatty acid pattern was similar to those of closely related *Nonomuraea* type strains, *N. candida* DSM 45086^T^ and *N. gerenzanensis* DSM 100948^T^. The morphological and chemotaxonomic properties of strain FMUSA5-5^T^ revealed characteristics that are typical of the genus *Nonomuraea*^3^. The 16S rRNA gene sequence of strain FMUSA5-5^T^ was first calculated for the 16S rRNA gene similarity using EzBiocloud server and demonstrated that strain FMUSA5-5^T^ shared the highest 16S rRNA gene sequence similarity to *N. candida* HMC10^T^ (98.8%), and *N. aridisoli* KC333^T^ (98.8%), followed by *N. gerenzanensis* ATCC 39727^T^ (98.7%). In contrast, the taxonomic position of strain FMUSA5-5^T^ in NJ, MP, and ML trees formed a distinct clade with all closest relatives, *N. candida* HMC10^T^, *N. aridisoli* KC333^T^, and *N. gerenzanensis* ATCC 39727^T^ (Figs. S2–S4). To confirm the taxonomic position of strain FMUSA5-5^T^, the genome-based taxonomy was analyzed. The draft genome size of strain FMUSA5-5^T^ consists of 12.4 Mbp (187 contigs and N50 of 187.1 kbp) with a genomic G + C content of 71.5%. The genome contains 11,583 predicted genes, including 11,142 protein-coding genes, 63 tRNA genes, and 2 rRNA genes (one 16S and one 23S) and exhibited approximately 31 biosynthetic gene clusters and was rich in biosynthetic gene clusters for terpenes, type I polyketide synthase, and nonribosomal peptide synthases (NRPS) (Tables S3, S4). Other genomic features of strain FMUSA5-5^T^ and related type strains were summarized in Table S5. The genome of strain FMUSA5-5^T^ and its closely related type strains, *N. candida* NRRL B-24552^T^, *N. aridisoli* KC333^T^, *N. gerenzanensis* L70^T^, were found to contain the clusters of type I polyketide synthase (T1PKS), type II polyketide synthase (T2PKS), type III polyketide synthase (T3PKS), NRPS, terpene, LAP, and NI-siderophore. By comparing the results of *N. candida* NRRL B-24552^T^, strain FMUSA5-5^T^ was not found to have lanthipeptide-class i, lanthipeptide-class ii, ranthipeptide, lipolanthine, and thioamitides gene clusters. Compared with *N. aridisoli* KC333^T^, strain FMUSA5-5^T^ was not found to have the clusters of lanthipeptide-class i, and thiopeptide. In contrast, the beta-lactam, ectoine, and butyrolactam gene clusters were only detected in the genome of *N. gerenzanensis* L70^T^ (Fig. 2). A codon tree based on multilocus sequence alignment of 100 conserved single-copy genes, which were detected among closely related *Nonomuraea* genomes publicly available on the autoMLST server, showed the same result compared with the phylogenomic tree obtained from TYGS that the taxonomic position of strain FMUSA5-5^T^ formed a distinct phylogenomic line with *N. candida* NRRL B-24552^T^, *N. aridisoli* KC333^T^, and *N. gerenzanensis* LT70^T^, implying that strain FMUSA5-5^T^ was different species of these closest neighbors (Fig. 3 and Fig. S5). Furthermore, average nucleotide identity (ANI), average amino acid identity (AAI), and digital DNA–DNA hybridization (dDDH) values between strain FMUSA5-5^T^ and its three closest type strains were in the range of 82.8–88.3% for ANI, 79.4–87.3% for AAI, and 29.5–38.5% for dDDH, which were lower than the recommended threshold values for prokaryotic species delineation (Table S6)^9,10^. These genome-based taxonomic details revealed the taxonomic position of strain FMUSA5-5^T^ that it was a different species of *N. candida* NRRL B-24552^T^, *N. aridisoli* KC333^T^, and *N. gerenzanensis* LT70^T^ and could be considered to represent a new species of the genus *Nonomuraea*. Although the genome-based taxonomic data presented that strain FMUSA5-5^T^ was a different species of three closest *Nonomuraea* type strains, however, the comparative phenotypic study between strain FMUSA5-5^T^ and its closest neighbors should be performed. It was shown that the phenotypic traits of strain FMUSA5-5^T^ were clearly different from its closely related *Nonomuraea* type strains, *N. candida* JCM 15928^T^**,** *N. aridisoli* DSM 107062^T^, *N. gerenzanensis* DSM 100948^T^ (Table 1). The significant differential phenotypic properties that distinguished strain FMUSA5-5^T^ from all closely related neighbors were the color of substrate mycelium, the spore surface ornamentation, and the utilization of inulin, L-cysteine, and L-tyrosine. The ability of strain FMUSA5-5^T^ to hydrolyze starch and utilization of D-melezitose could be used to discriminate it from *N. candida* JCM 15928^T^**,** and *N. gerenzanensis* DSM 100948^T^, while strain FMUSA5-5^T^ could differentiate from *N. aridisoli* DSM 107062^T^ in term of urea hydrolysis, the utilization of D-arabinose, D-raffinose, sucrose and the decomposition of hypoxanthine. These phenotypic traits demonstrate that strain FMUSA5-5^T^ were different species of *N. candida* JCM 15928^T^, *N. aridisoli* DSM 107062^T^, *N. gerenzanensis* DSM 100948^T^. It is concluded from phenotypic, chemotaxonomic, and genotypic properties that strain FMUSA5-5^T^ represents a new taxonomic status in the genus *Nonomuraea* as a novel species, and the name, *Nonomuraea composti* sp. nov. is proposed for the strain.

**Table 1:** Phenotypic features of strain FMUSA5-5^T^ compared to its related type strains.

Characteristics	Strain FMUSA5-5^T^	*N. candida* JCM 15928^T^	*N. aridisoli* DSM 107062^T^	*N. gerenzanensis* DSM 100948^T^*
Colour of substrate mycelium on ISP 2	Dark reddish brown	Light yellow	Orange brown	Orange brown
Spore surface ornamentation	Hairy	Smooth	Smooth	Rough
Maximum NaCl tolerance (%w/v)	1	3	4	3
Temperature range for growth (°C)	20–45	20–45	25–45	20–45
The pH range for growth	6–8	6–8	6–11	6–10
Milk peptonization	+	−	+	+
Nitrate reduction	+	−	w	+
Starch hydrolysis	−	+	−	+
Urea hydrolysis	−	−	+	+
Carbon utilization (1.0% w/v)				
D-Arabinose	+	+	−	+
D–Cellobiose	+	w	−	+
Inulin	+	−	−	−
*myo*-Inositol	−	+	−	−
D-Mannose	+	−	+	+
D-Melezitose	−	+	−	+
D-Raffinose	+	+	−	+
L-Rhamnose	+	+	+	+
Sucrose	+	w	−	+
Trehalose	+	w	w	NR
D-xylose	+	−	+	+
Nitrogen utilization (1.0% w/v)				
L-Cysteine	−	+	+	+
Glycine	−	+	−	−
L–Histidine	+	w	+	−
L-Phenylalanine	+	w	+	+
L-Serine	+	+	+	+
L-Valine	+	+	+	+
Decomposition (1.0% w/v) of				
Hypoxanthine	−	−	+	+
L–Tyrosine	−	+	+	+
Xanthine	−	−	−	−

All other phenotypic data were determined in this study. + , Positive; − , Negative; w, Weakly positive.

*Data taken from Saygin et al.^67^, and Dalmastri et al.^68^. NR, not reported.

### The secondary metabolite production by strain FMUSA5-5^T^

**Figure 4:**
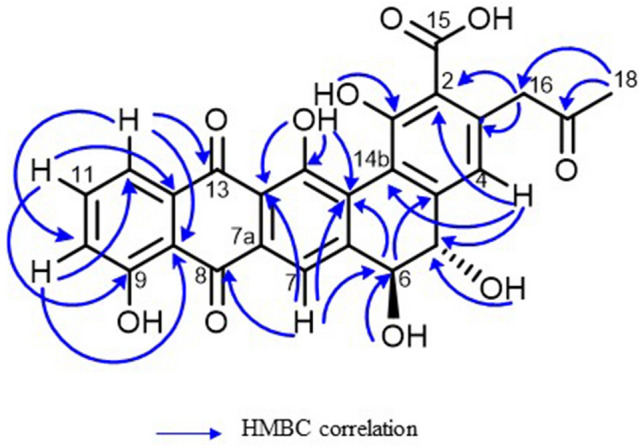
Key HMBC correlations of compound **1**.

**Figure 5:**
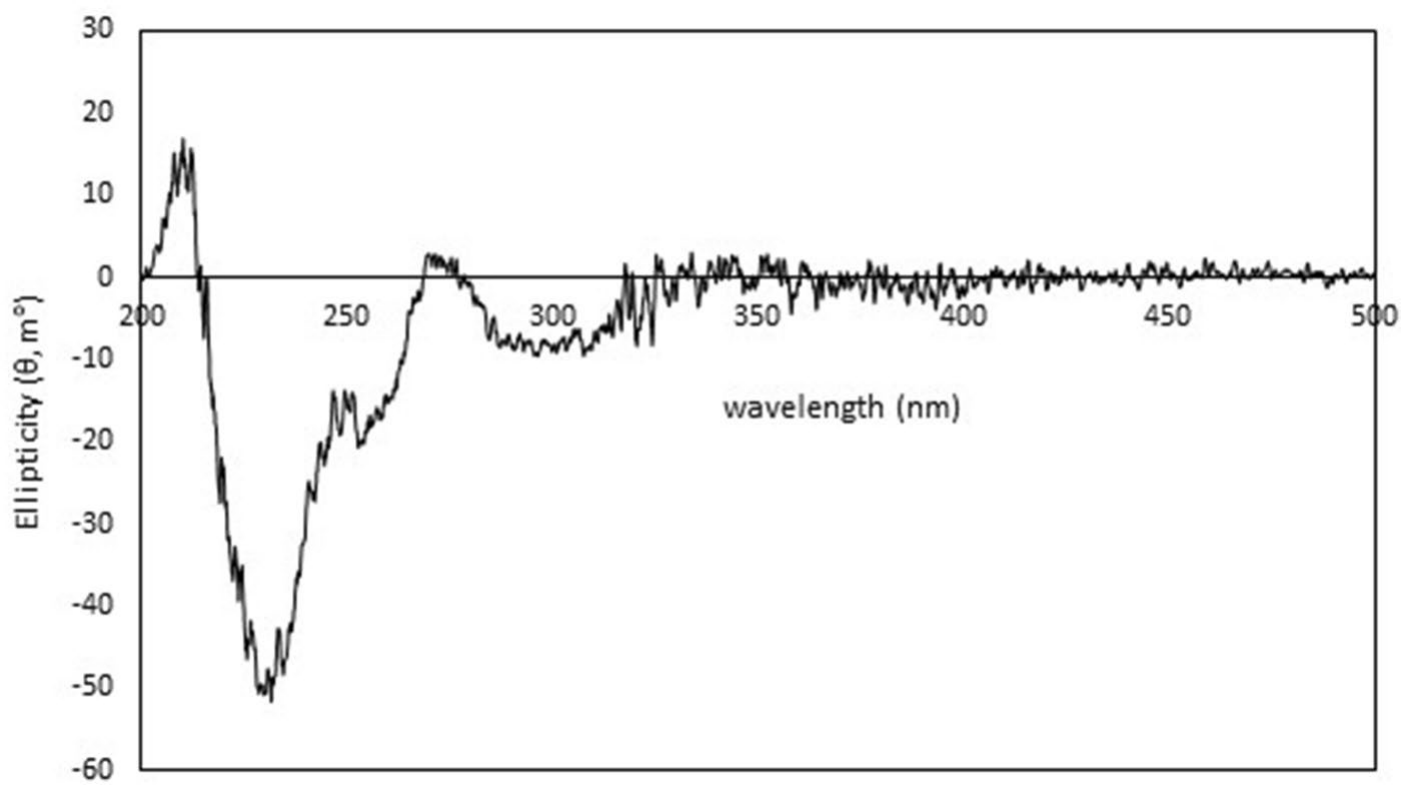
CD spectrum of compound **1**.

**Figure 6:**
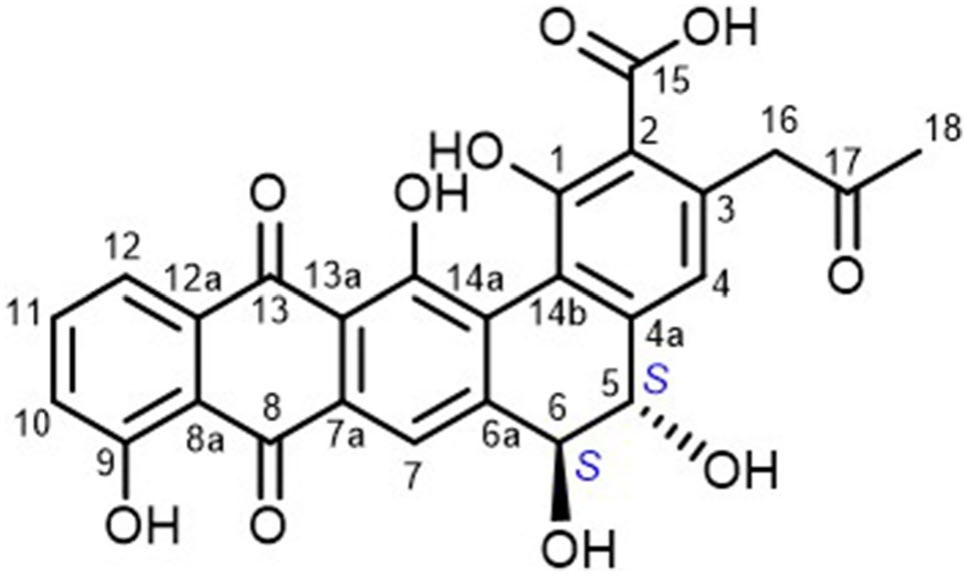
Chemical structure of compound **1**.

Due to many biosynthetic genes were detected in the genome of strain FMUSA5-5^T^, implying that strain FMUSA5-5^T^ has the potential to produce new substances; therefore, strain FMUSA5-5^T^ has received attention in the search for biologically active substances. Thus, the crude extract from the culture broth (ISP 2 medium) of strain FMUSA5-5^T^ was first screened for antimicrobial activity, and it exhibited potent antimicrobial activity against *B. cereus* ATCC 11778 and *S. aureus* ATCC 29213. The crude extract was continued for isolation and purification process using several chromatographic techniques. After that, the purified compound (compound **1**) was identified using spectroscopic techniques. Compound **1** was obtained as a dark red solid and insoluble in CHCl_3_, acetone, and sparingly soluble in MeOH. HRESIMS spectral data showed quasimolecular ion at *m/z* 489.0828 [M − H]^−^, suggesting the molecular formula of C_26_H_18_O_10_. The molecular formula indicated 18 degrees of unsaturation. The ^1^H NMR spectrum (Table 2) showed signals of a singlet methyl at δ_H_ 2.16, a set of non-equivalent methylene at δ_H_ 4.02 (d, *J* = 16.5 Hz) and 4.14 (d, *J* = 16.5 Hz), two sp^3^ methines at δ_H_ 4.21 (dd, *J* = 11.0, 5.1 Hz) and 4.32 (dd, *J* = 11.0, 5.0 Hz), and five aromatic methines at δ_H_ 6.83 (s), 7.29 (dd, *J* = 8.3, 1.1 Hz), 7.78 (dd, *J* = 8.3, 7.5 Hz), 7.65 (dd, *J* = 7.5, 1.0 Hz), and 8.09 (s). In addition, the ^13^C NMR spectrum (Table 2) showed extra 16 non-protonated carbons. The cross-peak correlations of H-10/H-11/H-12 in the COSY spectrum suggested the presence of 1,2,3-trisubstituted benzene. Also, the cross-peak correlation between H-5 and H-6 was observed in COSY spectrum and its large coupling constant of 11.0 Hz indicated *trans-*diaxial relationship. The HMBC spectrum showed correlations from H-10 to C-8a (δ_C_ 115.7) and C-12 (δ_C_ 118.5); from H-11 to C-9 (δ_C_ 160.9) and C-12a (δ_C_ 135.5); and from H-12 to C-10 (δ_C_ 122.0), C-8a, and C-13 (δ_C_ 181.2), together with the non-protonated carbon at δ_C_ 188.75, indicating a naphthalene-1,4-dione unit. Moreover, the HMBC spectrum (Fig. 4) showed correlations from H-4 to C-2 (δ_C_ 115.7), C-5 (δ_C_ 71.6), C-14b (δ_C_ 119.1), and C-16; from H-5 to C-4a (δ_C_ 144.1) and C-6 (δ_C_ 72.1); from H-6 to C-4a (δ_C_ 144.1) and C-6a (δ_C_ 146.5); and from H-7 to C-6, C-14a (δ_C_ 130.7), C-13a (δ_C_ 120.0), and C-8 (δ_C_ 188.8), linking a dihydrophenanthrene unit with a naphthalene-1,4-dione unit. Furthermore, the HMBC correlations from H_3_-18 to C-16 (δ_C_ 50.5) and C-17 (δ_C_ 205.2); and from H_2_-16 to C-2 (δ_C_ 115.7), C-3 (δ_C_ 140.6), C-4 (δ_C_ 117.3), and C-17 indicated a 2-propanone unit at C-3 and the HMBC correlations from 14-OH (δ_H_ 13.94) to C-14a, C-14 (δ_C_ 158.8), and C-13a; from 6-OH (δ_H_ 6.02) to C-6; from 5-OH (δ_H_ 5.80) to C-5 (δ_C_ 71.6) and from 1-OH (δ_H_ 17.79) to C-1 (δ_C_ 169.5) indicated the positions of hydroxyl groups at C-14, C-6, C-5 and C-1, respectively. According to the molecular formula, the remaining non-protonated carbon at δ_C_ 164.2 was placed at C-2 as a carboxylic group. The absolute configurations at C-5 and C-6 were assigned as *S* and *S*, respectively, since the CD spectrum of compound **1** (Fig. 5) showed negative Cotton effect at λ 235 nm and positive Cotton effect at λ 208 nm, the same pattern as those reported for pradimicin A^11^. Thus, chemical structure of compound **1** with the absolute stereochemistry was demonstrated as shown in Fig. 6. Pradimicin U is a trivial name for compound **1**. Pradimicin U could be categorized in pradimicin or benanomicin families that contain dihydrobenzo[α]naphthacenequinone skeleton. Compounds having dihydrobenzo [α]naphthacenequinone aglycone structure were biosynthesized by condensation of an acetyl unit with multiple malonyl-Co A units to form a polyketide backbone, which underwent regiospecifically cyclized by aromatase and cyclase enzymes involving type II polyketide synthase (PKS type II). The structures could be modified by oxidation, reduction, methylation, and glycosylation steps to provide the final polyketide complex structures^12^. Pradimicin U was closely related to the pradimicin molecule without extending the glycoside unit and amino acid into the molecule. The two hydroxyl groups with opposite stereochemical configurations at C-5 and C-6 were composed by P450 hydroxylases^13^. By analyzing the genome of strain FMUSA5-5^T^, we found that the biosynthetic gene clusters (BGCs) of pradimicin U were similar to the BGCs of pradimicin family, including two core biosynthetic genes, *pdmA* and *pdmB*, and six additional biosynthetic genes, *pdmC*, *pdmD*, *pdmG*, *pdmF*, *pdmK*, and *pdmT*, which responsible for synthesizing the dihydrobenzo[α]naphthacenequinone skeleton^12^ (Fig. S6). However, the BGCs of pradimicin U lacked two glycosyltransferase genes, *pdmS* and *pdmQ*, which are presumptively responsible for introducing the sugar moieties during pradimicin biosynthesis^14^. Based on these findings, the chemical structure of pradimicin U (Fig. 6) is likely to be a pradimicin family structure but lacks the sugar moiety.

**Table 2:** ^1^H and ^13^C NMR assignment of compound **1.**

**Position**	^**1**^**H NMR** δ_H_, multiplicity (*J* in Hz)	^**13**^**C NMR** δ_C_, type
1		169.5, C
2		115.7, C
3		140.6, C
4	6.83, s	117.3, CH
4a		144.1, C
5	4.21, dd (11.0, 5.1)	71.6, CH
6	4.32, dd (11.0, 5.0)	72.1, CH
6a		146.5, C
7	8.09, s	113.9, CH
7a		131.9, C
8		188.8, C
8a		115.7, C
9		160.9, C
10	7.29, dd (8.3, 1.1)	122.0, CH
11	7.78, d (8.3, 7.5)	137.0, CH
12	7.65, dd (7.5, 1.0)	118.5, CH
12a		135.5, C
13		181.2, C
13a		120.0, C
14		158.8, C
14a		130.7, C
14b		119.1, C
15		164.2, C
16	4.02, d (16.5) / 4.14, d (16.5)	50.5, CH_2_
17		205.2, C
18	2.16, s	29.9, CH_3_
	5.80, d (5.1)	
	6.02, d (5.0)	
	12.62, s	
	13.94, s	
	17.79, s

### Biological activities of the isolated compound (Pradimicin U)

The isolated compound **1** (pradimicin U) was subjected to evaluation for biological activity, including anti-multidrug-resistant *Plasmodium falciparum* K-1, antitubercular against *Mycobacterium tuberculosis* H37Ra, antibacterial against *B. cereus*, *S. aureus*, *Acinetobacter baumannii*, antifungal against *Alternaria brassicicola* activities and for cytotoxicity against cancerous (MCF-7, NCI–H187) and non-cancerous (Vero) cells. Pradimicin U exhibited a broad spectrum of biological activities such as antimalarial activity (against *P. falciparum*, K1 strain) with IC_50_ value of 3.65 µg/mL, anti-TB activity (against *M. tuberculosis* H37Ra) with MIC value of 25.0 µg/mL, anti-plant pathogenic fungal activity (against *A. brassicicola*) with MIC value of 25.0 µg/mL, and antibacterial activity (against Gram-positive bacteria such as *B. cereus* and *S. aureus*) with MIC values of 6.25 and 1.56 µg/mL, respectively. In addition, pradimicin U possessed strong anti-NCI-H187 activity with IC_50_ value of 5.69 µg/mL, while cytotoxicity against MCF-7 and Vero cells was very weak (IC_50_ values of 52.49 and 21.84 µg/mL, respectively). However, pradimicin U was inactive against Gram-negative bacteria such as *A. baumannii* at the maximum tested concentration. Furthermore, the antioxidant activity of pradimicin U was evaluated using 2,2-diphenyl-1-picrylhydrazyl (DPPH) free radical method. This assay is a widely used antioxidant assay to evaluate the radical scavenging activity of actinobacterial secondary metabolites. This method is based on the ability of antioxidants to donate hydrogen atoms to the stable free radical DPPH^15^. Pradimicin U displayed DPPH free radical scavenging activity with IC_50_ value of 52.1 µg/mL. In this study, we found that pradimicin U showed better antioxidant activity than butylated hydroxytoluene (BHT) (IC_50_ = 69.4 µg/mL), a positive control (Table S7). This is because pradimicin U has many hydroxy groups and conjugated double bonds in the molecule. It is known that hydroxy groups and conjugated double bonds can donate electrons to free radicals^16^.

Pradimicins A–C were originally isolated from the culture broth of *Actinomadura hibisca* P157-2 (ATCC 53557)^12^ and many analogs were later isolated from *Actinomadura* spp. and several mutant strains of *Actinomadura verrucosospora* subsp. *neohibisca*^17,18^. Generally, pradimicins and benanomicins were effective for antifungal activity^19–22^. Many *Nonomuraea* spp. have been reported as fascinating sources for the discovery of novel bioactive compounds. Several novel secondary metabolites from *Nonomuraea* spp. have been validated as outstanding antimicrobial substances against various pathogens. Thus, chemicals contained in the culture broth of *Nonomuraea* spp. must play an important role as bioactive compounds by inhibiting the growth of pathogens. These compounds include anthraquinones, phenols, alkaloids, terpenes, etc.^23^ For example, nonomuric acid, 3-hydroxy deoxydaunorubicinol aglycone, Ɛ-rhodomycinone and 7-deoxy-13-dihydrocarminomycinone, were isolated from the culture broth of *Nonomuraea rhodomycinica* NR4-ASC07^T^ and showed the antimicrobial activity against *P. falciparum*, K1 strain, *M. tuberculosis* H37Ra, and *B. cereus*^24^. In 2017, Nazari et al.^25^ reported kistamicin, the glycopeptide antibiotics, produced from *Nonomuraea* sp. ATCC 55,076. This compound possessed antiviral activity. Madurahydroxylactone was isolated from the culture broth of the terrestrial *Nonomuraea* sp. AN100570. This compound showed an inhibitory effect on the growth of *S. aureus* by inhibiting FtsZ protein with an IC_50_ value of 53.4 µM. Moreover, this compound could inhibit the growth of multidrug-resistant *Staphylococcus aureus* (MRSA) with a MIC value of 1 µg/mL^26^. Hypogeamicin A, a S-bridged pyronaphthoquinone dimer, and hypogeamicin B-D are the outstanding secondary metabolites, which were isolated from the terrestrial *Nonomuraea* sp. Hypogeamicin A exhibited a strong cytotoxic activity against colon cancer (TCT-1 cell line) with IC_50_ = 6.4 µM while hypogeamicin B-D displayed antibacterial activity against *Bacillus subtilis* with MIC ranged from 7–28 µg/mL^27^. From the information presented above, it can be concluded that *Nonomuraea* spp. are an important microorganism for the production of new secondary metabolites and can be further applied in biotechnological and pharmaceutical use in the near future.

### Description of *Nonomuraea composti *sp. nov.

#### *Nonomuraea composti* (com.pos′ti. N.L. gen. n. *composti* of compost)

Cells are Gram-stain-positive and aerobic. It grows well on ISP 2, ISP 3, and nutrient agar. Grows moderately on ISP 4, ISP 5, ISP 7, and Czapek’s sucrose. The poorly growth is observed on ISP 6. Dark reddish brown substrate mycelium is observed on ISP 2, ISP 3, and nutrient agar. Aerial spore masses is produced on ISP 2 for 30 days of cultivation. The clusters of rod-shaped spores with hairy surface are detected. Reddish brown diffusible pigments are found on ISP 2, ISP 4, ISP 5, and nutrient media. The reduction of nitrate, milk peptonization and production of catalase are positive. Negative results are observed for oxidase activity, liquefaction of gelatin, hydrolysis of starch, hydrogen sulfide production, and urease production. Decomposes adenine, but not cellulose, hypoxanthine, L-tyrosine and xanthine. Utilizes L-arabinose, D-cellobiose, D-fructose, D-galactose, D-glucose, inulin, D-lactose, D-mannitol, D-mannose, D-melibiose, L-rhamnose, D-ribose, sucrose, D-trehalose, and D-xylose; but does not utilize dextran, *myo*-inositol, D–melezitose, D-raffinose, and xylitol as sole carbon sources. Utilizes L-arginine, L-asparagine, L-histidine, 4-hydroxyproline, L-methionine, L-phenylalanine, L-proline, L-serine, L-threonine, and L-valine; but does not utilize DL-2-aminobutyric acid, and L-cysteine as sole nitrogen sources. The growth temperature is between 20–45 °C. Maximum NaCl for growth is 1% (w/v). The pH range for growth is 6–8. Cell wall peptidoglycan contains *meso*-diaminopimelic acid. The major menaquinones are MK-9(H_4_) and MK-9(H_2_), while MK-9(H_6_) and MK-9(H_0_) are minor component. Glucose, mannose, ribose, and madurose are detected as whole-cell sugars. The phospholipid profile contains phosphatidylethanolamine, phosphatidylmethylethanolamine, phosphatidylglycerol, phosphatidylinositol mannoside, three phosphoglycolipids, and five unidentified phospholipids. The major fatty acids (> 10%) are *iso*-C_16:0_, 10-methyl C_17:0_, C_16:0_. The DNA G + C content of the type strain is 71.5%. The type strain, FMUSA5-5^T^ (= TBRC 8481^T^ = NBRC 113443^T^), is an actinomycete isolated from the bio–fertilizer of *Musa* spp. collected from Suphanburi province, Thailand. The GenBank accession number for the 16S rRNA gene sequence of strain FMUSA5-5^T^ is LC377946. The whole-genome shotgun project has been deposited at GenBank under the accession JAATEP000000000.

## Conclusions

This study reports the discovery of novel species of the genus *Nonomuraea* and its promising secondary metabolite. *Nonomuraea* sp. FMUSA5-5^T^ was taxonomically characterized using a polyphasic approach, including genome-based taxonomic analysis. The strain is judged as a novel species of the genus *Nonomuraea*, and the name *Nonomuraea* *composti* sp. nov. is proposed. The crude extract from the culture broth of *N.* *composti* sp. nov. displayed interesting biological activity and led to the isolation of a new secondary metabolite, namely pradimicin U. This promising compound showed a broad spectrum of biological activities such as antimalarial, anti-TB, anti-Gram-positive pathogenic bacteria, as well as cytotoxic activity against human cancerous cells. This finding suggests that *N.* *composti* sp. nov. is a valuable microbial resource, and its secondary metabolite could be applied for further biotechnological and pharmaceutical proposes.

## Experimental procedures

### Isolation, cultivation, and preservation of strain FMUSA5-5^T^

Strain FMUSA5-5^T^ was isolated from a bio-fertilizer of *Musa* spp. collected from Suphanburi province, Thailand (14° 38′ 71″ N and 100° 14′ 69″ E). The isolation was done according to the protocol of Duangupama et al.^28^ with minor modifications. Briefly, the sample was air-dried at 30 °C for 7 days and heated at 100 °C for 30 min. The sample was serial diluted (1000-fold) to 10^–3^ with 0.01% sterile SDS in distilled water and spread onto soil extract agar (1 g soluble starch, 0.1 g KNO_3_, 0.005 g FeSO_4_.7H_2_O, 0.005 g MgSO_4_.7H_2_O, 0.001 g CaCl_2_ 0.2H_2_O, 1.5 g agar, 100 ml soil extract solution; pH 7.2) supplemented with antibiotics suggested by Thawai et al.^29^. After 21 days of incubation at 30 °C, a purplish-brown colony of strain FMUSA5-5^T^ was picked and purified on yeast extract-malt extract agar (International *Streptomyces* Project, ISP 2 medium)^30^. The pure culture was maintained in glycerol solution (20%, v/v) at − 80 °C for short-term preservation or lyophilized for long-term preservation.

### Polyphasic taxonomic characterizations

#### Morphological, cultural, physiological, and biochemical characteristics

To determine the morphology property, the 30 days-old of strain FMUSA5-5^T^ was observed by scanning electron microscopy (JSM-6610 LV; JEOL). Cultural characteristics were determined using International *Streptomyces* Project (ISP) media 1–7 by cultivation for 14 days at 30 °C. The colors of the aerial and substrate mycelia and diffusible pigments were used for determining using the ISCC-NBS color charts^31^. Temperature (10–50 °C), NaCl tolerance (0–10% w/v), and pH (4–12 at intervals of 1 pH units) range for growth were tested in ISP 2 broth for 14 days. The various phenotypic traits such as the utilization of sole nitrogen sources, decomposition of unsoluble compounds (adenine, hypoxanthine, xanthine, tyrosine, and cellulose), hydrolysis of starch, nitrate reduction, milk peptonization, and gelatin liquefaction were tested using the standard method of Arai^32^, Williams and Cross^33^, and Gordon et al.^34^. The utilization of sole carbon sources (1%, w/v) was performed using the protocol described by Supong et al.^35^. The closest reference strains, *Nonomuraea candida* DSM 45086^T^, *Nonomuraea aridisoli* DSM 107062^T^ were cultured under the same conditions for comparative analyses.

#### Chemotaxonomic analyses

In this study, the typical chemotaxonomic properties of actinomycete taxonomy were characterized using standard methods. To prepare the dried cells of strain FMUSA5-5^T^, the method of Phongsopitanun et al.^36^ was used. The isomer of diaminopimelic acid, reducing sugars in cell hydrolysates, and type of menaquinones were determined using the previous methods described by Hasegawa et al.^37^, Komagata and Suzuki^38^, Minnikin et al.^39^, and Collins et al.^40^, respectively. To analyze the polar lipid pattern of strain FMUSA5-5^T^, the dried cells were extracted and determined according to the protocol of Minnikin et al.^8^. Cellular fatty acid profile was analyzed using gas chromatography (model 6890; Agilent) according to the instructions of the Microbial Identification System (MIDI)^41,42^.

#### Genome-based taxonomy and genome analysis for secondary metabolite production

The genomic DNA used for PCR amplification and whole-genome sequencing was extracted using the method described by Tamaoka^43^. The 16S rRNA gene amplification was carried out using the primers 9F and 1541R^44^. The experimental condition was followed by the suggestion of Kittiwongwattana et al.^45^. The 16S rRNA gene sequence similarity of strain FMUSA5-5^T^ was calculated using the EzBioCloud server^46^. The 16S rRNA gene sequence of strain FMUSA5-5^T^ and the 16S rRNA gene sequences of the closely related type strains of *Nonomuraea* species obtained from EzBioCloud database were used for the construction of the 16S rRNA gene trees using the MEGA X software^47^. Various types of 16S rRNA gene trees, neighbor-joining^48^, maximum-parsimony^49^, and maximum-likelihood^50^ trees were analyzed in this study. The stability of the clades in the trees was analyzed by bootstrap analysis with 1000 resamplings^51^. The genomic DNA was sent to Chulalongkorn University, Thailand for whole-genome sequencing using an Illumina Miseq platform (Illumina) (2 × 200 bp paired-end reads). The NCBI Prokaryotic Genome Annotation Pipeline (PGAP) was used for genome annotation. The essential genome-based taxonomic traits, the average nucleotide identity (ANI), average amino acid identity (AAI), and digital DNA–DNA hybridization (dDDH) values were calculated using the online platforms, the Jspecies^52^, the Kostas Lab AAI calculator^53^, and the genome-to-genome distance calculator (GGDC 2.1; blast + method)^54^. To construct a high-resolution species tree using genomic data from multiple loci, an automated multi-locus species tree (autoMLST) pipeline (https://automlst.ziemertlab.com/)^55^ was used. The final autoMLST tree was reconstructed using the maximum-likelihood algorithm using MEGA X, with *Corynebacterium lipophiloflavum* DSM 44291^T^ (GCA_000159635) as an outgroup. To confirm the taxonomic position of strain FMUSA5-5^T^, the phylogenomic tree constructed by the Type (strain) Genome Server (TYGS, https://tygs.dsmz.de/) was also performed^56^. To predict possible secondary metabolites and predictive biosynthetic gene cluster (BGC) for the biosynthesis of the novel compound, pradimicin U, isolated from strain FMUSA5-5^T^, the genome sequence was annotated using antiSMASH 7.0^57^ with Known ClusterBlast, ActiveSiteFinder, ClusterBlast, Cluster PFam analysis, and SubClusterBlast and manually searched based on the compound chemical structure.

#### General chemical experimental procedures

CD spectrum was performed in MeOH using a J-810 (JASCO) spectropolarimeter. Optical rotation was measured using a JASCO P-2000 digital polarimeter. The UV spectrum was recorded in MeOH using a V-730 UV–Vis spectrophotometer (JASCO). FTIR spectrum was recorded using a Bruker ALPHA spectrometer. NMR experiment was performed on a Bruker Avance III™ HD 500 MHz NMR spectrometer. HRESIMS data was acquired using a Bruker MicrOTOF mass spectrometer.

#### Fermentation, extraction, and isolation of a bioactive substance

An actinomycete strain FMUSA5-5^T^ was cultivated on ISP 2 agar at 30 °C for 5 days. The agar was cut into pieces (1 × 1 cm^2^) and inoculated into a 250 ml Erlenmeyer flask (10 flasks), containing 100 mL ISP 2 medium (composed of (% w/v): 0.4% glucose, 0.4% powdered yeast extract, and 1.0% powdered malt extract in distilled water at pH 7.2). The culture was incubated at 30 °C on a rotary shaker at 200 rpm for 6 days. Then, an equal volume (25 ml) of the seed culture was transferred into 40 × 1 L Erlenmeyer flasks, which each contained 250 mL of ISP 2 medium at pH7.2 and the production culture was cultivated at 30 °C on rotary shakers at 200 rpm. After 14 days, the whole culture was extracted three times with an equal volume of ethyl acetate (EtOAc). EtOAc was evaporated at reduced pressure to dryness to obtain a brown gum (0.71 g). The gum was precipitated with 100% MeOH and the solid was filtered through a Whatman No.1 membrane. The insoluble solid was discarded. The filtrate was then applied to a Sephadex LH-20 column (3.5 × 40 cm), eluted with 100% MeOH to give compound **1** (39.2 mg).

Compound **1**: Dark red amorphous solid, [α]^25^_D_ =  + 577.2 (*c* 0.01, DMSO); UV (CH_3_CN) λ_max_ (log ε) 214 (4.33), 244 (4.35), 329 (3.93), 467 (3.97) nm; CD (0.07 g/L in DMSO) λ (Δε) 208 (+ 3.15), 235 (− 10.06) nm; FTIR (ATR) ν_max_ 3430, 1698 (sh), 1623, 1597, 1473, 1456, 1374, 1268 cm^−1^; ^1^H (DMSO-*d*_*6*_, 500 MHz) and ^13^C NMR data (DMSO-*d*_*6*_, 125 MHz), see Table 2; HRESIMS *m/z* 489.0828 [M − H]^−^ (calcd for C_26_H_17_O_10_, 489.0827) (Fig. S7–S20).

#### Biological activity tests

The microculture radioisotope method^58^ was used for the in vitro quantitative assessment of antimalarial activity against *Plasmodium falciparum* (K1 strain, multidrug-resistant strain). Dihydroartemisinin and chloroquine were used as positive controls and showed IC_50_ values of 0.0025 µg/mL and 0.129 µg/mL, respectively. The green fluorescent protein microplate assay (GFPMA)^59^ was used for evaluation of antitubercular activity against *Mycobacterium tuberculosis* H37Ra and cytotoxicity against non-cancerous (Vero) cells (African green monkey kidney fibroblasts, ATCC CCL-81). Rifampicin, streptomycin, isoniazid, ofloxacin, and ethambutol were used as positive controls for antitubercular activity, while ellipticine was used as positive control for cytotoxicity. They exhibited MIC values of 0.013, 1.25, 0.09, 0.78, and 0.94 µg/mL, respectively and ellipticine showed IC_50_ value of 0.72 µg/mL. The optical density microplate assay^60,61^ was employed for the evaluation of antibacterial activity against both Gram-positive (*Bacillus cereus* ATCC 11778 and *Staphylococcus aureus* ATCC 29213) and Gram-negative (*Acinetobacter baumannii* ATCC 19606) bacteria. Positive controls for anti*-B. cereus* and anti-*S. aureus* activities were rifampicin and vancomycin, which had MIC values of 0.625, 0.781 and 4.1, 1.05 µg/mL, respectively*.* While positive controls for anti-*A. baumannii* activity were rifampicin and erythromycin, which had MIC values of 3.13 and 25.0 µg/mL. The 5(6)-carboxyfluorescein diacetate (CFDA) fluorometric detection^62–64^ was employed for evaluation of anti-*Alternaria brassicicola* (BCC 42724). Amphotericin B was used as a positive control and showed a MIC value of 0.781 µg/mL. The resazurin-based microplate assay (REMA)^65^ was applied for the evaluation of cytotoxicity against MCF-7 (human breast cancer, ATCC HTC-22) and NCI-H187 (human small-cell lung cancer, ATCC CRL-5804) cells. Positive controls for anti-MCF-7 activity were doxorubicin (IC_50_ 8.64 µg/mL) and tamoxifen (IC_50_ 8.06 µg/mL) and those for anti-NCI-H187 activity were doxorubicin (IC_50_ 0.056 µg/mL) and ellipticine (IC_50_ 3.62 µg/mL). Maximum tested concentrations were done at 100 µg/mL for all tests, except that for anti-malarial activity was done at 10 µg/mL. MIC values represented the lowest concentrations of tested compounds that could inhibit more than 90% bacterial or fungal growth. IC_50_ values showed concentrations that cause 50% cell death according to the dose–response curve, which is plotted between compound concentrations and % cell inhibition using the curve-fitting method. The antioxidant activity of the isolated compound was estimated using the method described by Supong et al.^66^ with minor modifications.

#### Accession number of ***Nonomuraea*** sp. FMUSA5-5^T^

The GenBank accession number for the complete 16S rRNA gene sequence of *Nonomuraea* sp. FMUSA5-5^T^ is LC377946. The Whole Genome Shotgun projects for *Nonomuraea* sp. FMUSA5-5^T^ has been deposited at GenBank under the accession JAATEP000000000. The strain is deposited in the Thailand Bioresource Research Center and NITE Biological Resource Center for code numbers TBRC 8481 and NBRC 113,443, respectively.

### Supplementary Information

Supplementary Information.

## Data Availability

The datasets generated and/or analyzed during the current study are available on the NCBI website and with the following accession codes at the NCBI database: *Nonomuraea* sp. FMUSA5-5^T^: LC377946 and JAATEP000000000.

## Abbreviations

ANI: Average nucleotide identity
AAI: Average amino*-*acid identity
dDDH: Digital DNA–DNA hybridization
DAP: Diaminopimelic acid
DSM: German Collection of Microorganisms and Cell Cultures GmnH
EtOAc: Ethyl acetate
FT-IR: Fourier transformation infrared spectroscopy
GC: Gas chromatography
ISP: International *Streptomyces* Project
GFPMA: Green fluorescent protein microplate assay
MeOH: Methanol
MS: Mass spectrometry
REMA: Resazurin-based microplate assay
NMR: Nuclear magnetic resonance
NRPS: Non-ribosomal peptide synthetase
RNAp: Ribonucleic acid polymerase
smBGCs: Secondary metabolites biosynthetic gene clusters
T1PKS: Type I polyketide synthase
T2PKS: Type II polyketide synthase
T3PKS: Type III polyketide synthase
TBRC: Thailand Bioresource Research Center
NBRC: NITE Biological Resource Center
