# Supplementary material for: Pradimicin U, a promising antimicrobial agent isolated from a newly found Nonomuraea composti sp. nov
Source: Sci Rep. 2024 May 13;14:10942. doi: 10.1038/s41598-024-60744-w (PMC11091084; doi:10.1038/s41598-024-60744-w)
Supplement: Supplementary file 1 — Supplementary Information. [file 41598_2024_60744_MOESM1_ESM.pdf]

Scientific Reports

Pradimicin U, a promising antimicrobial agent isolated from a newly found *Nonomuraea composti* sp. nov.

Thitikorn Duangupama[1], Pattama Pittayakhajonwut[2], Chakapong Intaraudom[2], Chanwit Suriyachadkun[3], Sarin Tadtong[4], Nattakorn Kuncharoen[5], Ya-Wen He[6], Somboon Tanasupawat[7], Chitti Thawai[1,8*]

Affiliation

[1]Department of Biology, School of Science, King Mongkut's Institute of Technology Ladkrabang, Bangkok 10520, Thailand

[2]National Center for Genetic Engineering and Biotechnology (BIOTEC), National Science and Technology Development Agency (NSTDA), 113 Thailand Science Park, Phaholyothin Road, Khlong Nueng, Khlong Luang, Pathum Thani, 12120, Thailand

[3]Thailand Bioresource Research Center (TBRC), National Center for Genetic Engineering and Biotechnology (BIOTEC), National Science and Technology Development Agency (NSTDA), 113 Thailand Science Park, Phaholyothin Road, Khlong Nueng, Khlong Luang, Pathum Thani, 12120, Thailand

[4]Department of Pharmacognosy, Faculty of Pharmacy, Srinakharinwirot University, Nakhon nayok 26120, Thailand

[5]Department Department of Plant Pathology, Faculty of Agriculture, Kasetsart University Bangkok 10900, Thailand

[6]State Key Laboratory of Microbial Metabolism, School of Life Sciences & Biotechnology, Shanghai Jiao Tong University, Shanghai 200240, P R China

[7]Department of Biochemistry and Microbiology, Faculty of Pharmaceutical Sciences, Chulalongkorn University, Bangkok, 10330, Thailand

[8]Actinobacterial Research Unit, School of Science, King Mongkut's Institute of Technology Ladkrabang, Bangkok 10520, Thailand

***Corresponding author**

Assoc. Prof. Dr. Chitti Thawai, Department of Biology, School of Science, King Mongkut's Institute of Technology Ladkrabang, Bangkok 10520, Thailand

E-mail: chitti.th@kmitl.ac.th   Phone: +66(2)3298400 ext 235   Fax: +66(2)3298427

Fig. S1. Polar lipid appearing on the two-dimensional thin-layer chromatograms of strain FMUSA5-5^{T}.

(A) Phosphomolybdic acid's TLC chromatogram; (B) Molybdenum blue spray's TLC chromatogram; (C) Ninhydrin's TLC chromatogram; (D) Anisaldehyde's TLC chromatogram; (E) Dragendorff's TLC chromatogram; Abbreviation: phosphatidylethanolamine (PE), phosphatidylmethylethanolamine (PME), phosphatidylglycerol (PG), phosphatidylinositol mannoside (PIM), phosphoglycolipid (PGL), and unidentified phospholipid (PL). The solvent systems used for development are as follows:

The 1st solvent system : Chloroform:MeOH:Water (65:25:4)

The 2nd solvent system : Chloroform:Acetic acid:MeOH:Water (40:7.5:6:2)

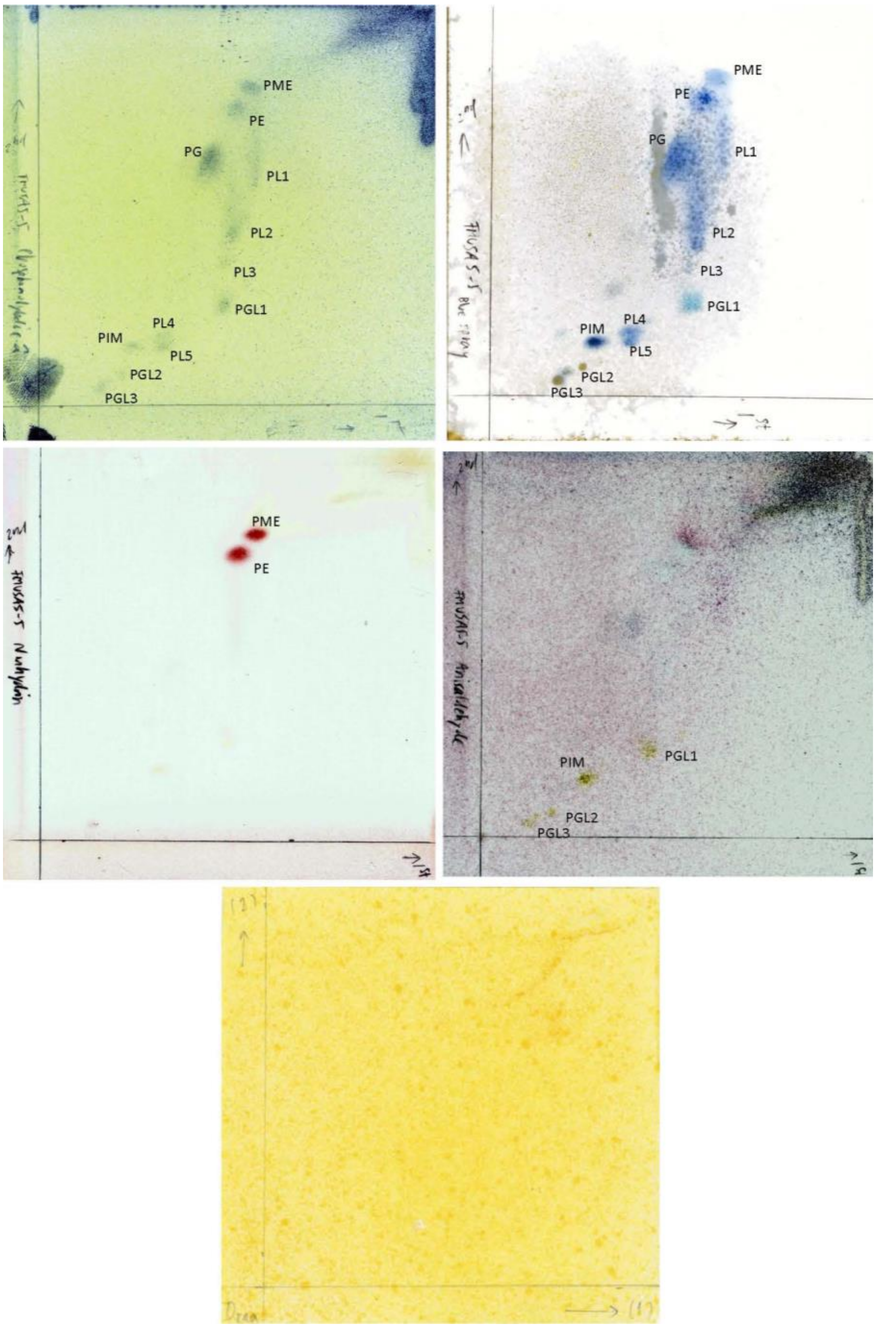

Fig. S2. Neighbor-joining tree based on 16S rRNA gene sequences showing a relationship between strain FMUSA5-5^{T} and related taxa. The numbers on the branches indicate the percentage bootstrap values of 1,000 replicates; only values ≥50 % are shown. Bar, 0.01 substitutions per nucleotide position.

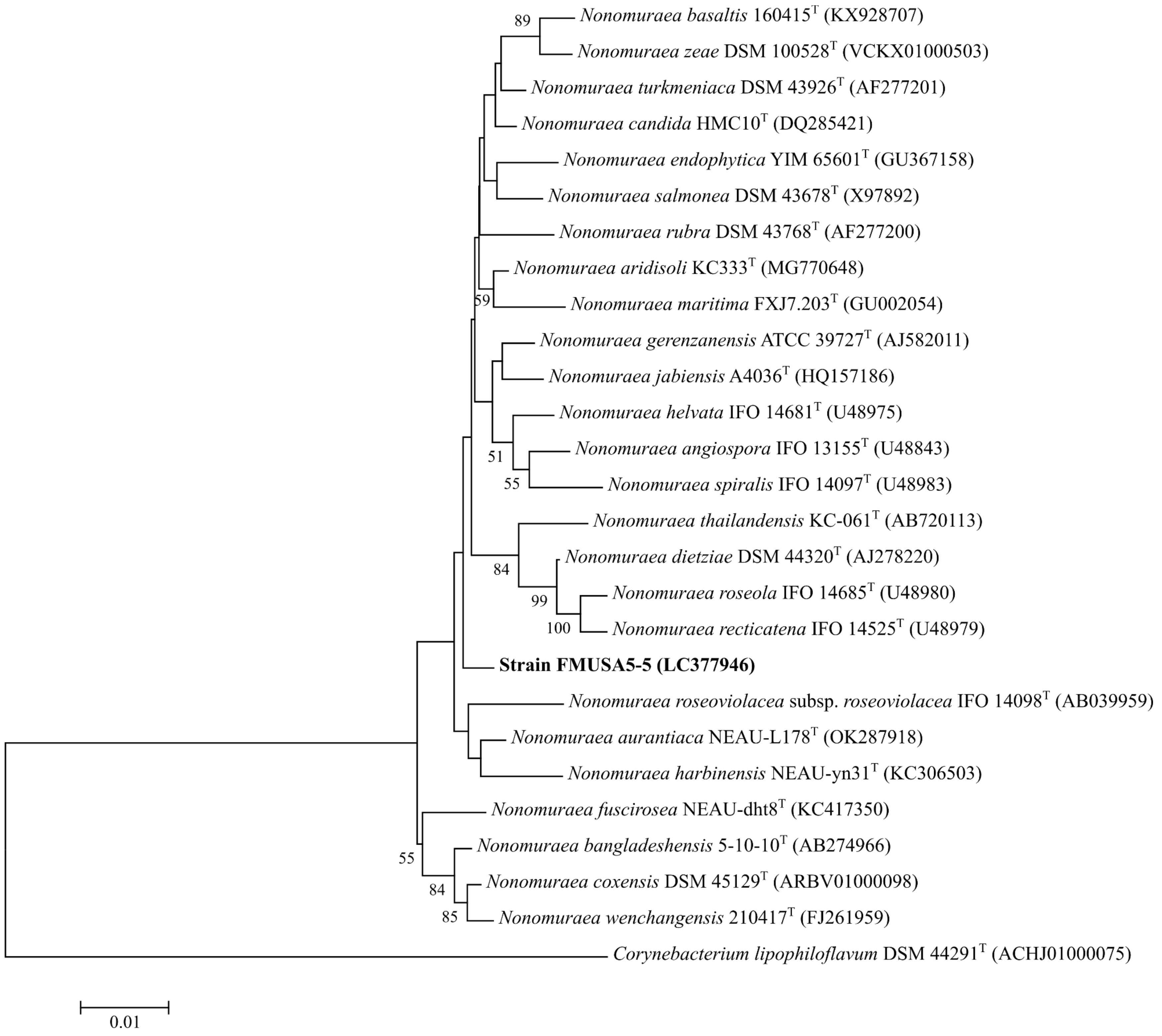

Fig. S3. Maximum parsimony tree based on 16S rRNA gene sequences showing a relationship between strain FMUSA5-5^T and related taxa. The numbers on the branches indicate the percentage bootstrap values of 1,000 replicates; only values ≥50 % are shown.

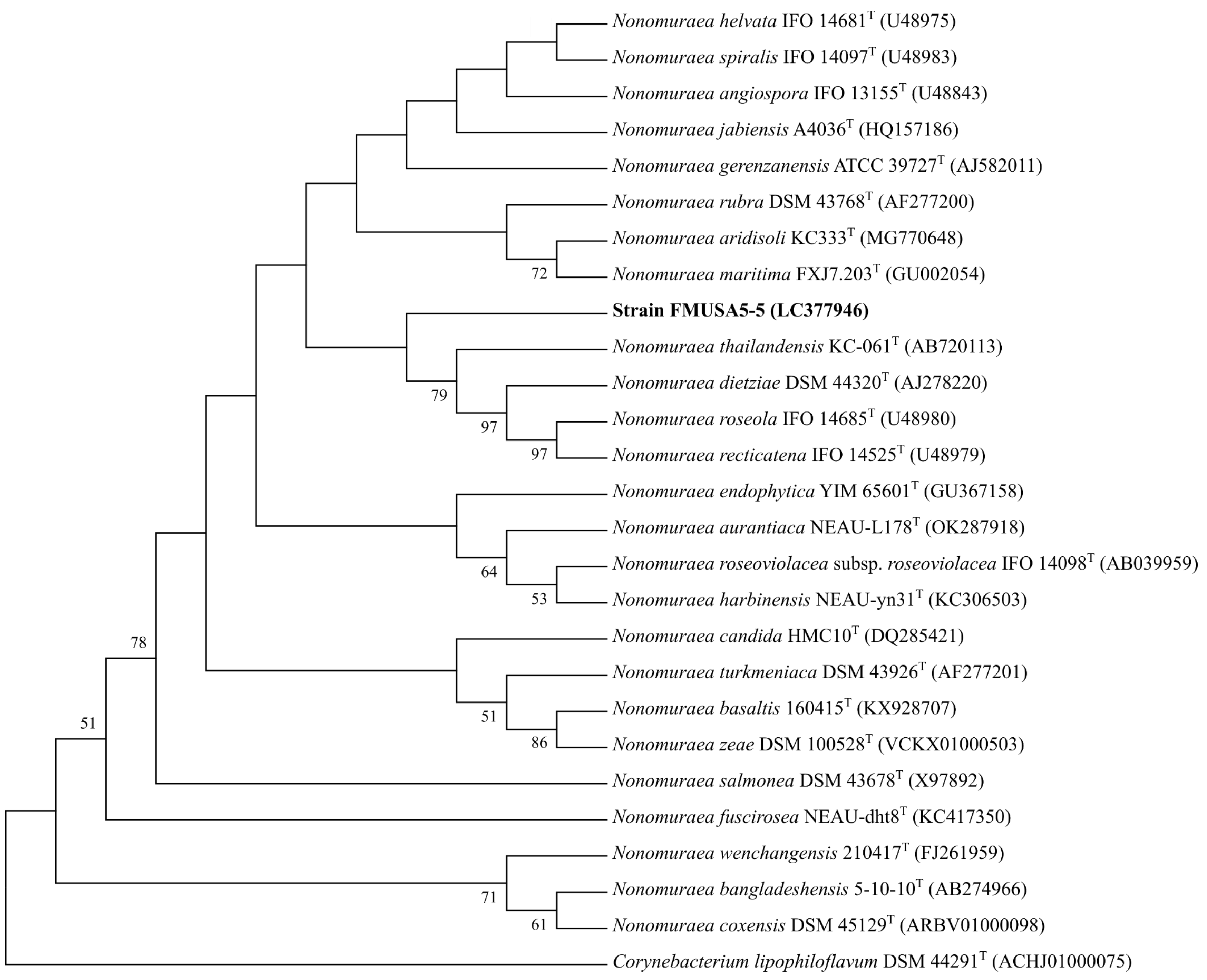

Fig. S4. Maximum-likelihood tree based on 16S rRNA gene sequences showing a relationship between strain FMUSA5-5^{T} and related taxa. The numbers on the branches indicate the percentage bootstrap values of 1,000 replicates; only values ≥50 % are shown. Bar, 0.01 substitutions per nucleotide position.

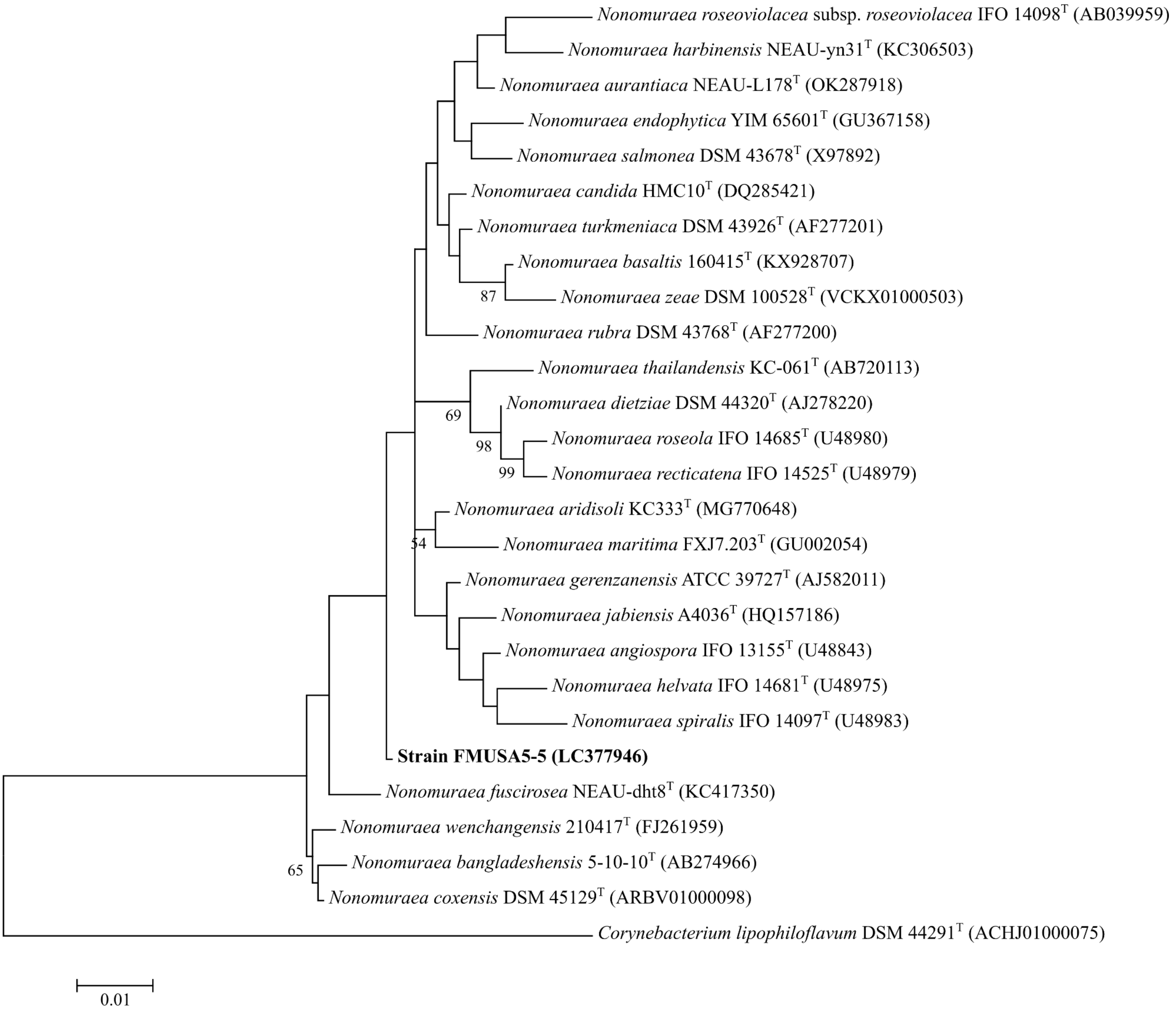

Fig. S5. The phylogenomic tree of strain FMUSA5-5^T and their related type strains of the genus *Nomuraea* obtained from TYGS. Tree inferred with FastME 2.1.6.1[70] from GBDP distances calculated from genome sequences. The branch lengths are scaled in terms of GBDP distance formula d_5. The numbers above branches are GBDP pseudo-bootstrap support values >60 % from 100 replications, with an average branch support of 87.6 %. The tree was rooted at the midpoint[71].

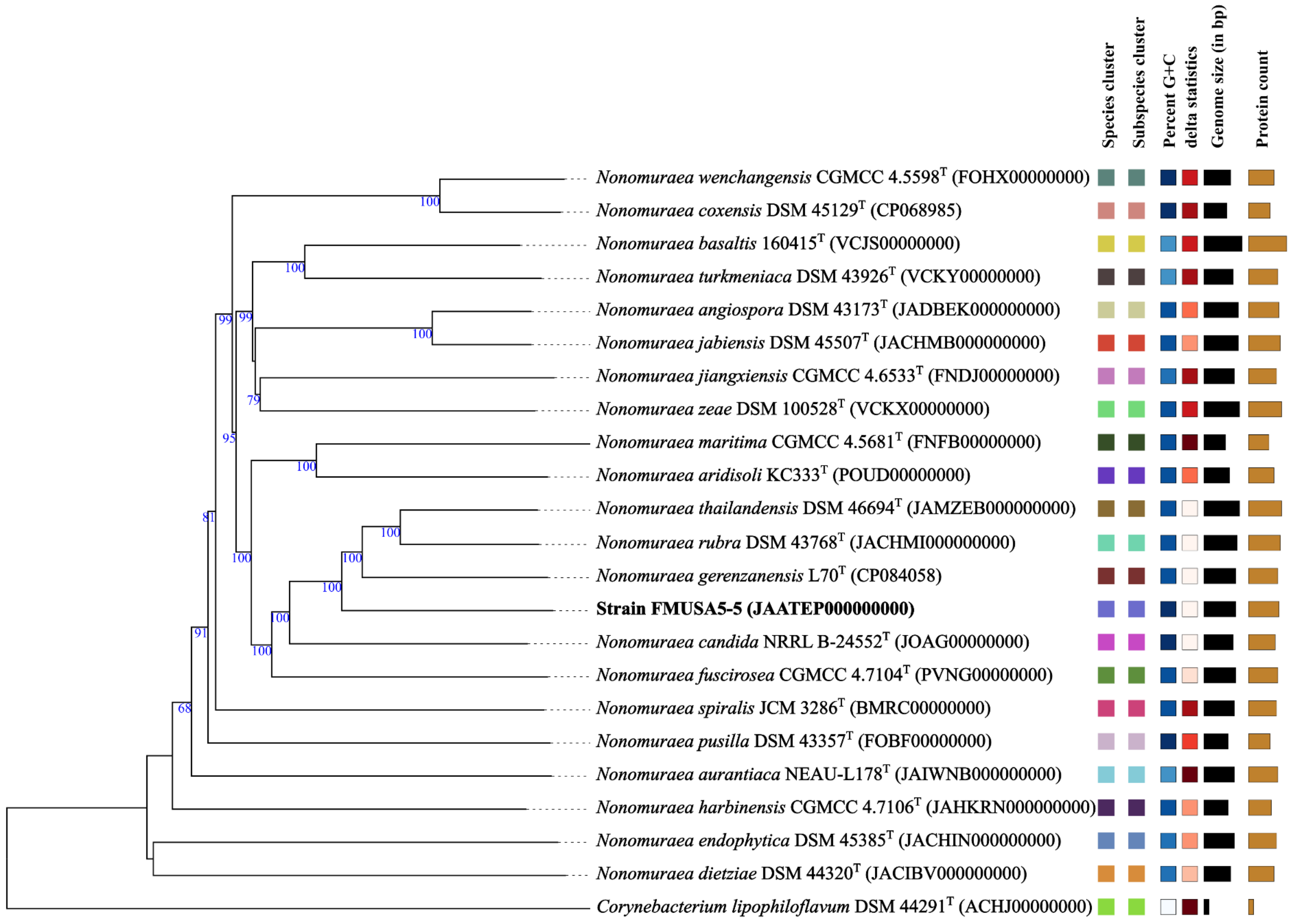

Fig. S6 Organization of the predicted putative biosynthetic gene cluster for pradimicin U in the genome of strain FMUSA5-5^T.

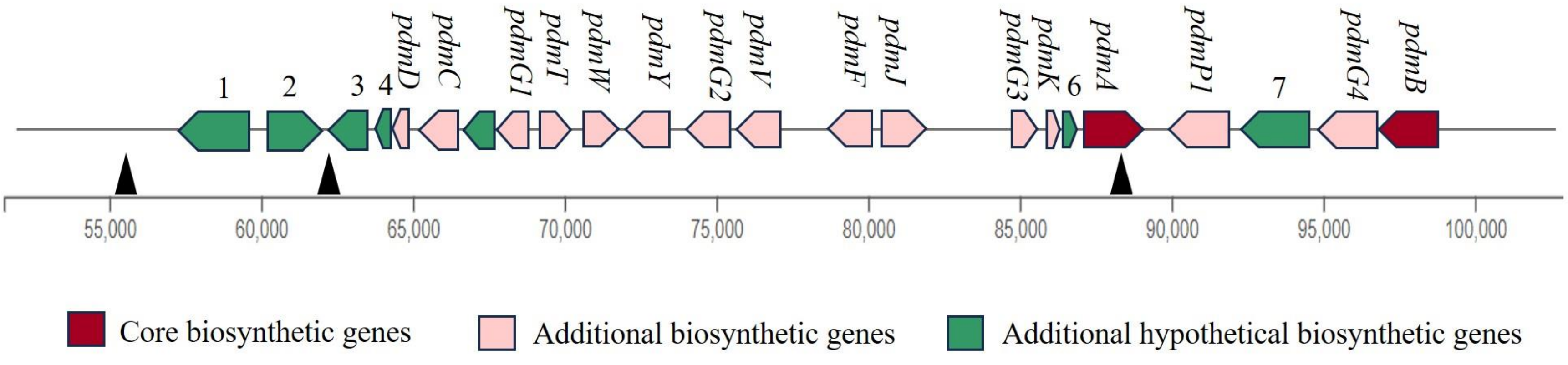

Fig. S7. ^{1}H NMR spectrum (500 MHz) of compound **1** in DMSO-*d6*

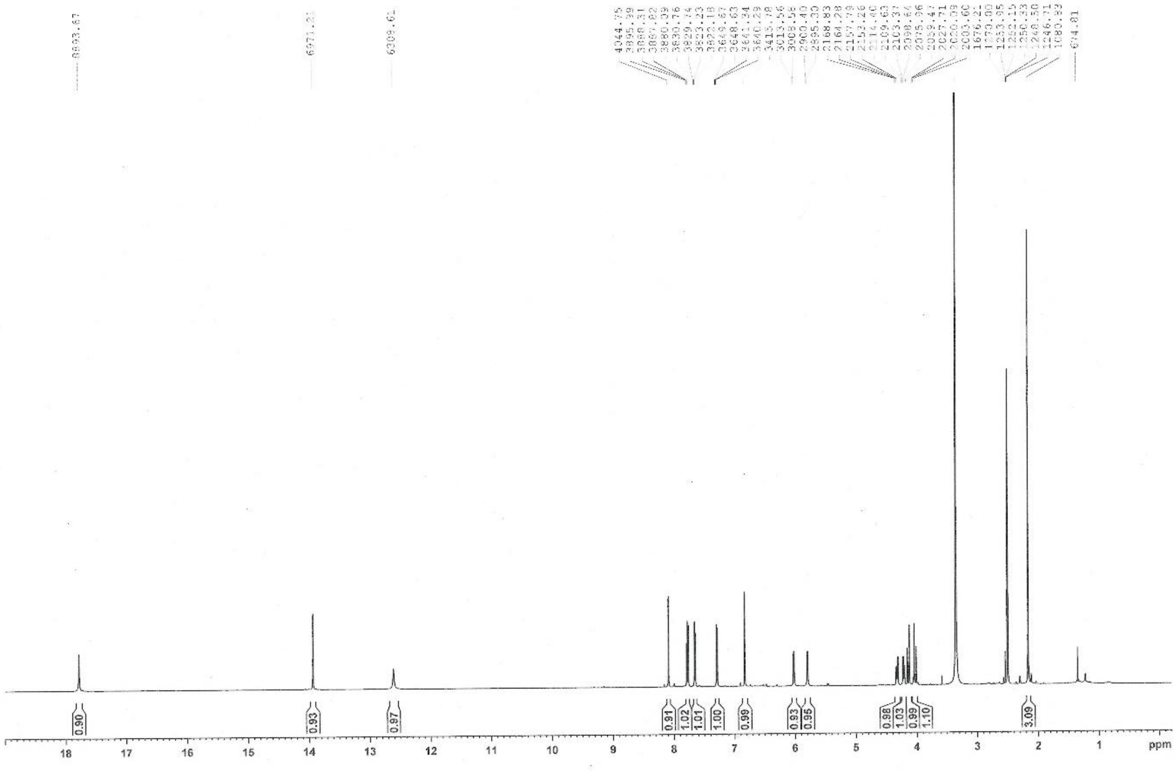

Fig. S8. ^{13}C NMR spectrum (125 MHz) of compound **1** in DMSO-*d6*

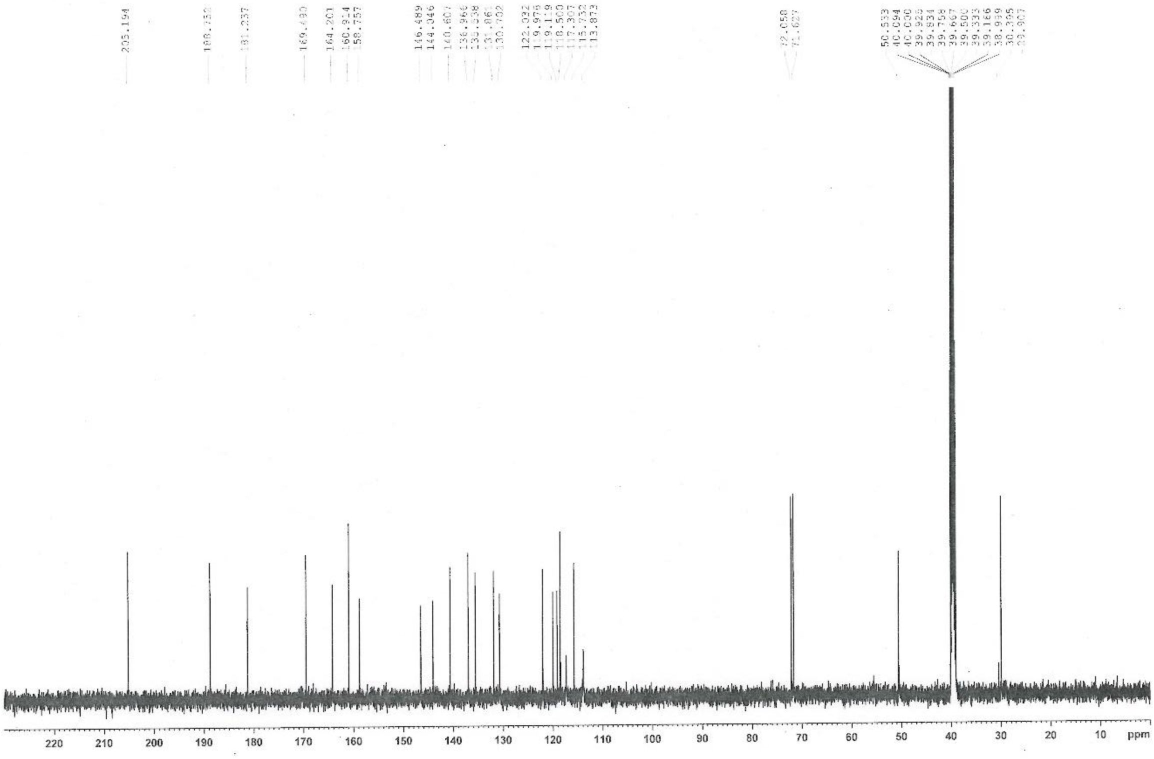

Fig. S9. ^{13}C NMR spectrum (expansion) of compound **1** in DMSO-d_6

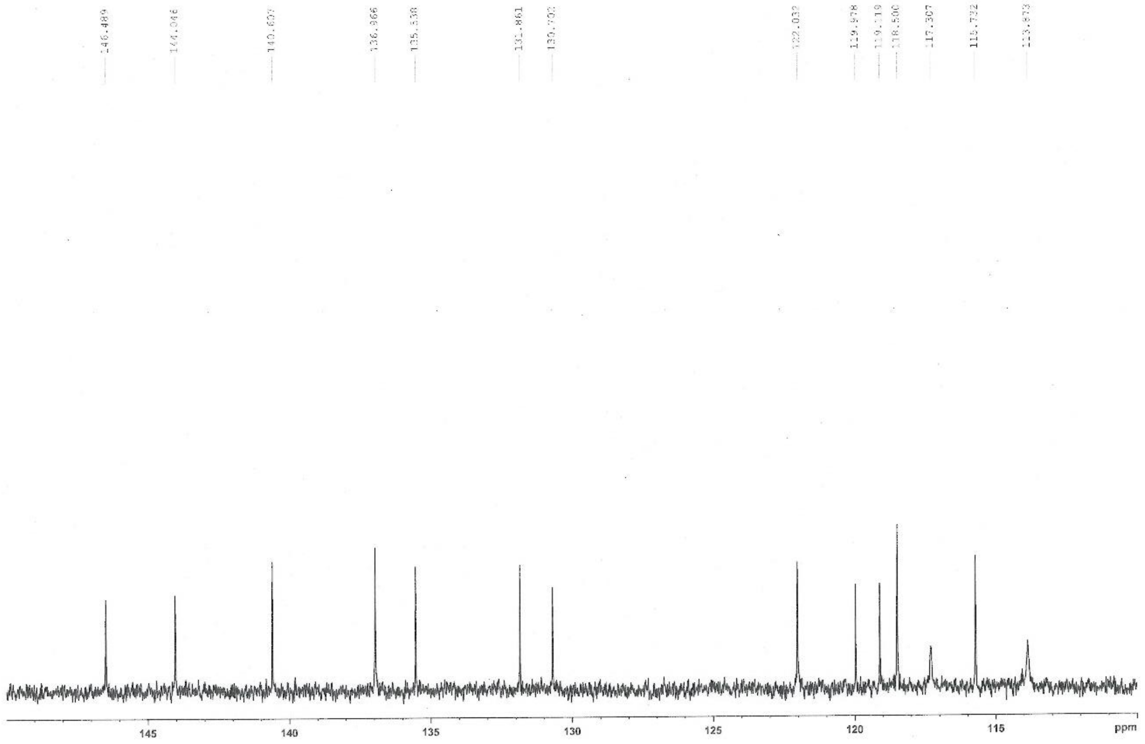

Fig. S10. ^{13}C NMR and DEPT-135 spectrum of compound **1** in DMSO-d_6

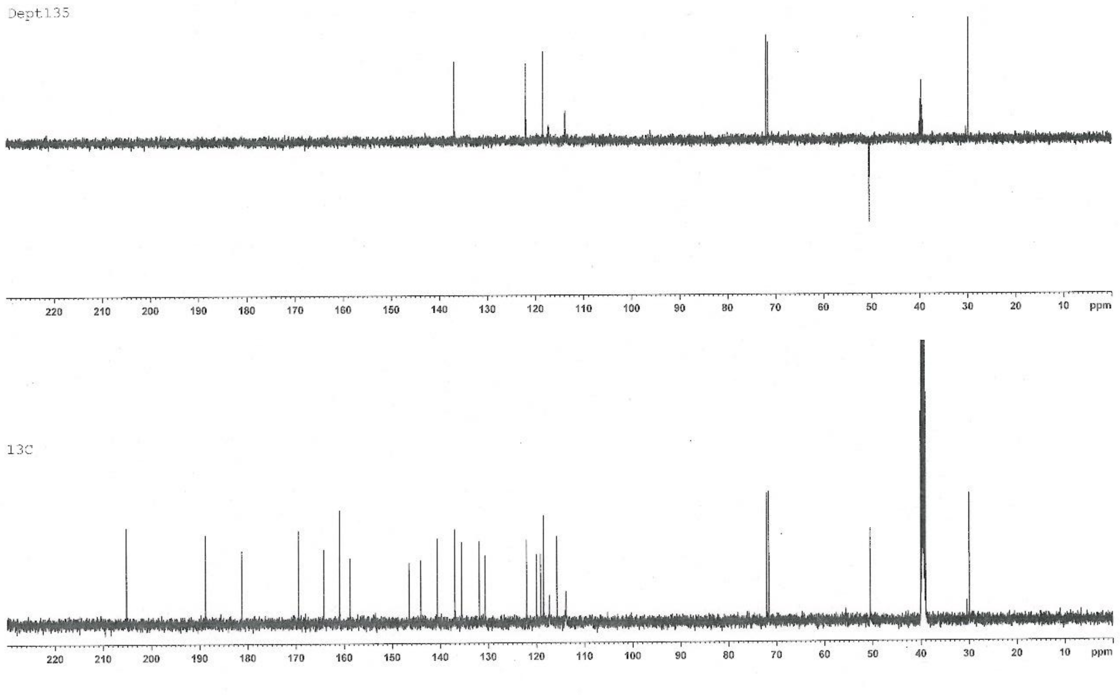

Fig. S11. HSQC spectrum of compound **1** in DMSO-d_6

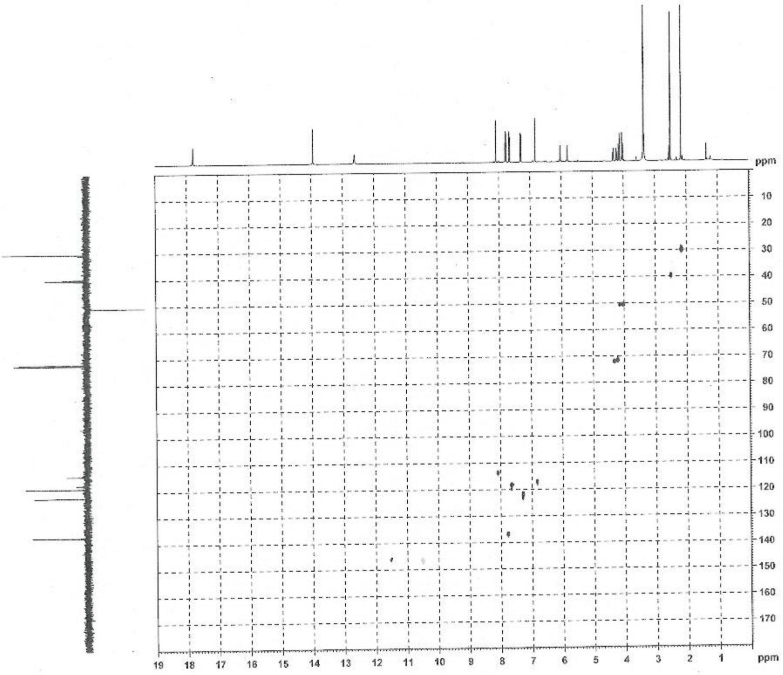

Fig. S12. HSQC spectrum (expansion) of compound **1** in DMSO-d_6

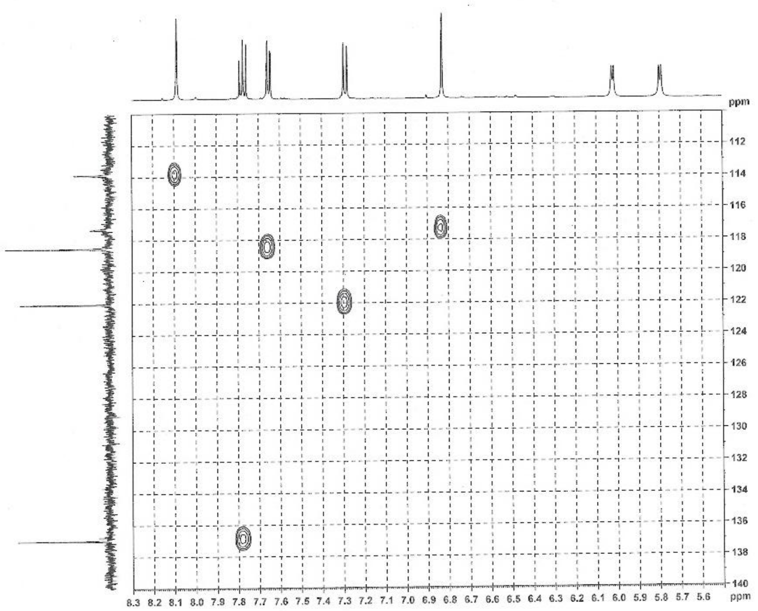

Fig. S13. COSY spectrum of compound **1** in DMSO-d_6

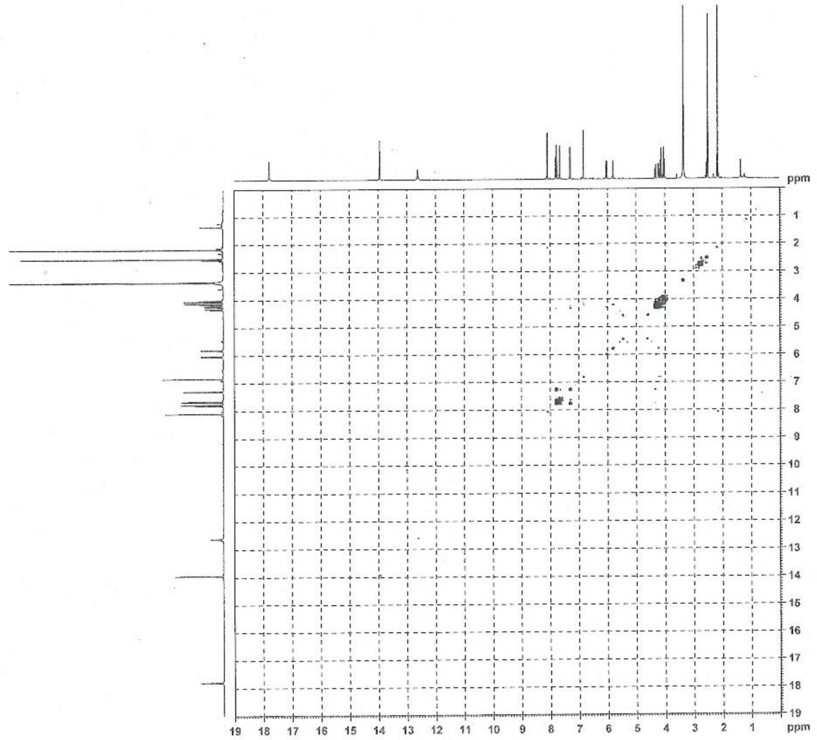

Fig. S14. COSY spectrum (expansion) of compound **1** in DMSO-d_6

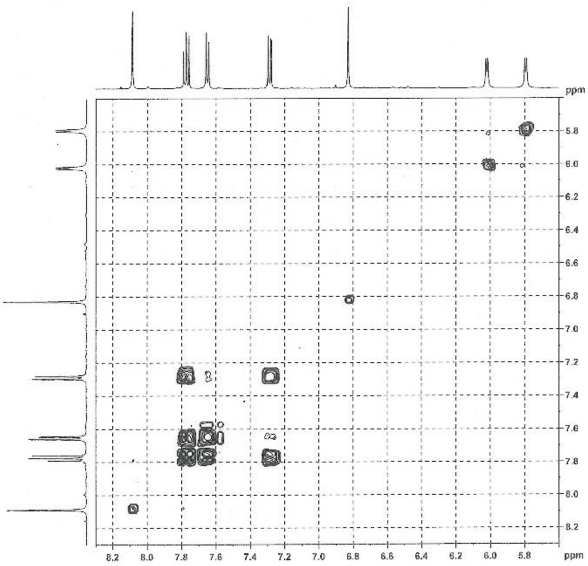

Fig. S15. HMBC spectrum of compound **1** in DMSO-d_6

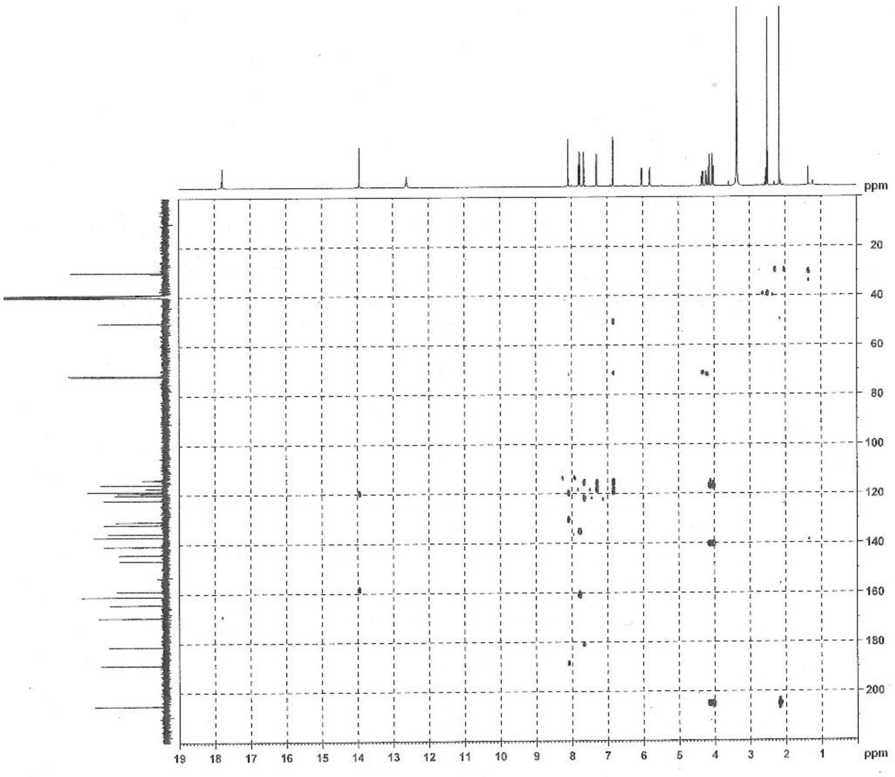

Fig. S16. HMBC spectrum (expansion) of compound **1** in DMSO-d_6

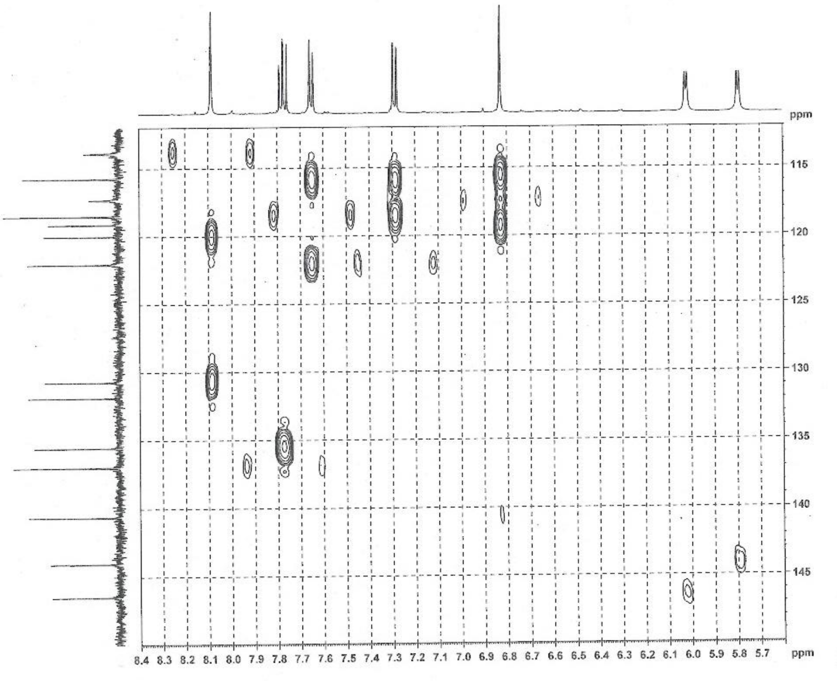

Fig. S17. HMBC spectrum (expansion) of compound **1** in DMSO-*d₆*

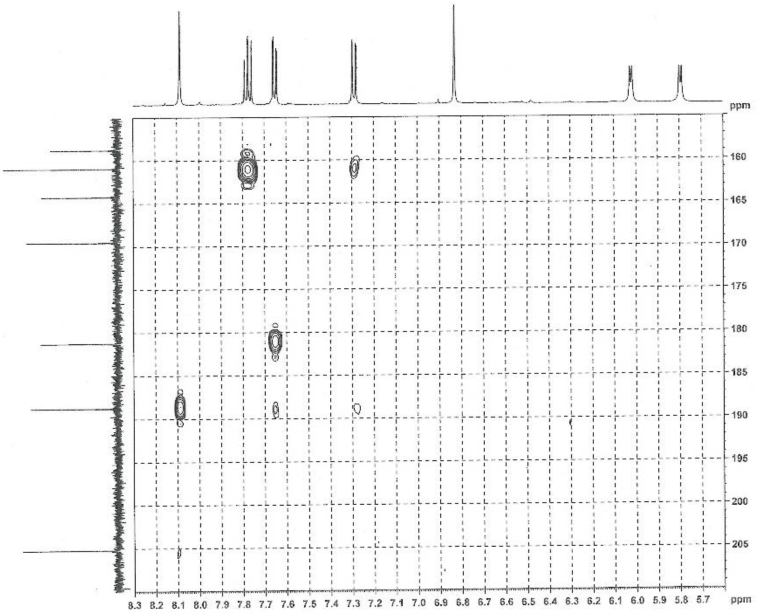

Fig. S18. HRESIMS spectrum of compound **1**

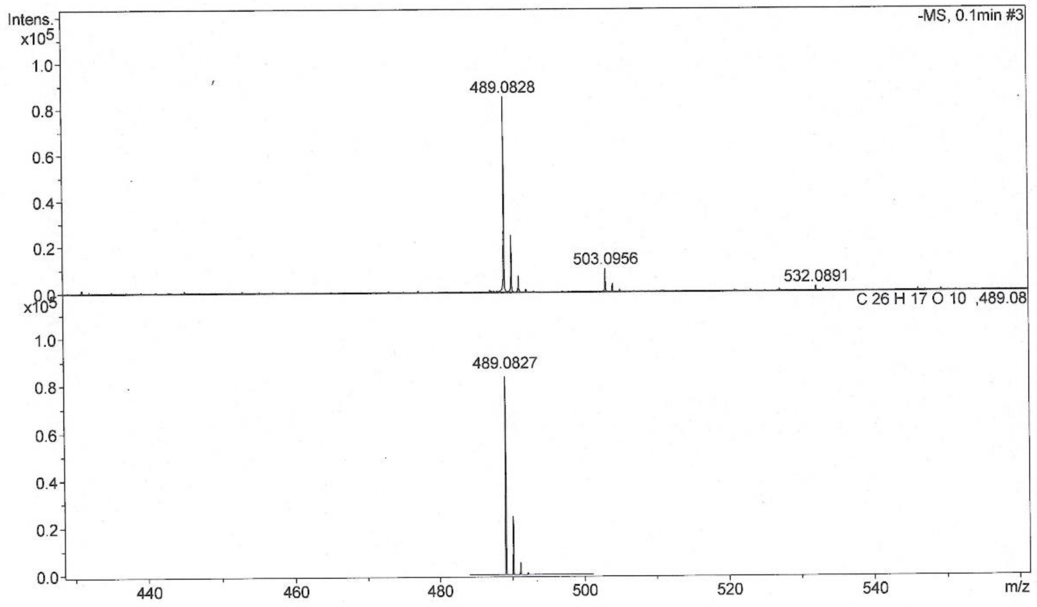

Fig. S19. FT-IR spectrum of compound **1**

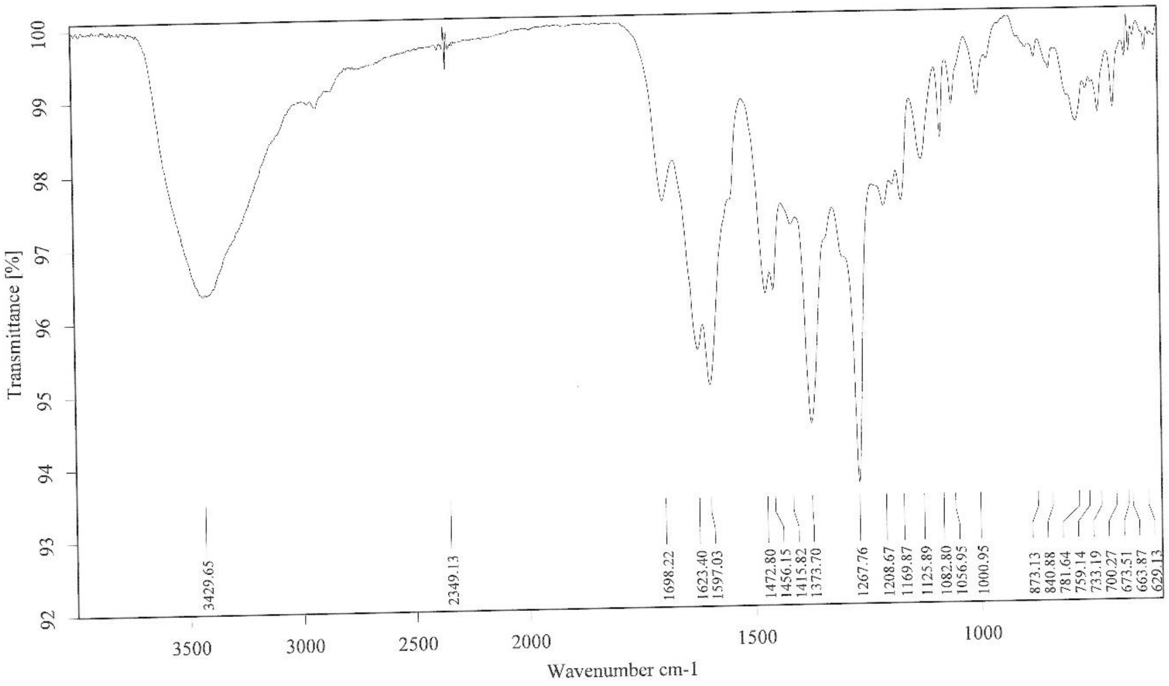

Fig. S20. UV spectrum of compound **1** (in CH_3CN)

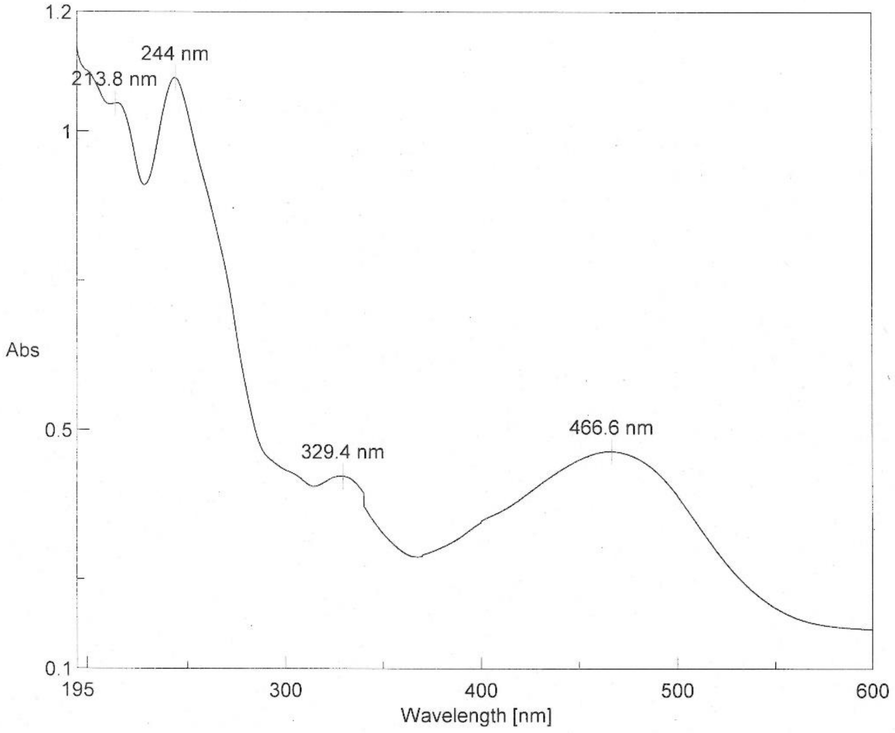

Table S1. Cultural characteristics of strain FMUSA5-5^{T} and the closest relatives.

*Data taken from Dalmastri et al.[69]

Strains	Media	Growth	Substrate mycelium	Aerial spore mass	Soluble pigment
FMUSA5-5^{T}	ISP 2	Good	Dark reddish brown	-	Reddish brown
	ISP 3	Good	Dark reddish brown	-	-
	ISP 4	Moderate	Reddish brown	-	Reddish brown
	ISP 5	Moderate	Brown	-	Reddish brown
	ISP 6	Poor	Brown	-	Pale brown
	ISP 7	Moderate	Dark brown	-	-
	Czapek's Sucrose	Moderate	Reddish brown	-	Light brown
	NA	Good	Dark reddish brown	-	Reddish brown
Nonomuraea candida JCM 15928^{T}	ISP 2	Good	Light Yellow	Pale Greenish Yellow	-
	ISP 3	Good	Moderate Yellow	White	-
	ISP 4	Poor	Yellowish White	-	-
	ISP 5	Good	Pale Yellow	-	-
	ISP 6	Poor	Pale Yellow	-	-
	ISP 7	Moderate	Pale Yellow	-	-
	Czapek's Sucrose	Moderate	Pale Yellow	-	-
	NA	Good	Strong Reddish Orange	Greyish Pink	-

Table S1. Cultural characteristics of strain FMUSA5-5^T and the closest relatives. (cont.)

*Data taken from Dalmastri et al.[69]

Strains	Media	Growth	Substrate mycelium	Aerial spore mass	Soluble pigment
N. aridisoli DSM 107062^T	ISP 2	Good	Orange-brown	White	-
	ISP 3	Good	Orange-brown	-	-
	ISP 4	Moderate	Yellowish white	White	-
	ISP 5	Moderate	Light brown	-	-
	ISP 6	Poor	Yellowish white	-	-
	ISP 7	Moderate	Light yellowish brown	-	-
	Czapek's Sucrose	Moderate	Light yellow	-	-
	NA	Good	Purplish brown	-	-
N. gerenzanensis DSM 100948^{T*}	ISP 2	Good	Beige	-	-
	ISP 3	Moderate	Light brown	White	Light brown
	ISP 4	Good	Brownish orange	-	-
	ISP 5	Good	Brownish orange	White	-
	ISP 6	Moderate	Brown	-	-
	ISP 7	Good	Brownish orange	White	-
	Czapek's Sucrose	Moderate	Dark cream	-	-
	NA	Good	Light beige	-	-

Table S2. Cellular fatty acid composition of strain FMUSA5-5^T and the closely related type strains.

The culture was grown in ISP 2 broth on a rotary shaker for five days at 30 °C.

* Data were taken from Ay[72].

**Fatty acid** (%)	Strain FMUSA5-5^T	*N. candida* JCM 15928^T	*N. aridisoli* DSM 107062^T	*N. gerenzanensis* DSM 100948^{T*}
$C_{12:0}$	0.5	-	-	-
$C_{13:0}$	0.4	-	0.1	-
iso-$C_{13:0}$	0.1	-	-	-
$C_{14:0}$	3.3	1.0	-	2.6
iso-$C_{14:0}$	0.7	-	-	-
$C_{15:0}$ 2OH	0.4	1.4	0.1	-
$C_{15:0}$ 3OH	-	-	-	1.1
anteiso-$C_{15:0}$	1.1	-	-	-
$C_{15:0}$	1.8	2.4	2.7	2.4
iso-$C_{15:0}$	6.3	4.2	8.7	4.5
$C_{15:1}$ $\omega 6$c	0.2	-	0.1	-
$C_{16:0}$	10.2	3.4	3.8	6.0
iso-$C_{16:0}$	19.4	38.1	42.8	20.0
anteiso-$C_{16:0}$	0.3	-	0.1	-
$C_{16:0}$ 2OH	1.4	1.1	-	3.0
iso-$C_{16:0}$ 2OH	-	7.1	13.1	8.3
iso-$C_{16:1}$G	3.0	2.3	6.2	4.2
$C_{16:1}$ $\omega 9$c	1.0	1.7	0.4	5.0
$C_{17:0}$	7.1	5.8	4.3	1.8
iso-$C_{17:0}$	1.9	-	-	1.5
anteiso-$C_{17:0}$	3.1	1.2	-	-
$C_{17:1}$ $\omega 8$c	3.5	-	0.1	-
$C_{17:1}$ $\omega 9$c	-	-	1.0	5.2
anteiso-$C_{17:1}$ $\omega 9$c	0.4	-	-	-
$C_{17:0}$ 2OH	0.5	-	-	-
$C_{18:0}$	4.6	0.2	0.1	-
iso-$C_{18:0}$	1.0	1.8	2.1	-
iso-$C_{18:1}$	0.2	-	0.1	-
$C_{18:1}$ $\omega 9$c	1.5	-	0.1	1.9
$C_{18:1}$ 2OH	0.4	-	-	-
$C_{19:0}$	-	-	0.1	-
10-methyl $C_{16:0}$	-	0.7	1.5	5.3
10-methyl $C_{17:0}$	15.8	18.7	11.8	15.7
10-methyl *iso*-$C_{18:0}$	-	6.8	-	2.6
10-methyl $C_{18:0,}$ TBSA	3.7	2.1	0.7	4.5
Summed feature 3	3.0	-	-	-
Summed feature 8	1.0	-	-	-
Summed feature 9	2.2	-	-	-

*Summed Features are fatty acids that cannot be resolved reliably from another fatty acid using the chromatographic conditions chosen. The MIDI system groups these fatty acids together as one feature with a single percentage of the total. Summed feature 3 contains $C_{16:1}\omega$7c and/or $C_{15:0}$ *iso* 2-OH; Summed feature 8 contains $C_{18:1}\omega$7c and/or $C_{18:1}\omega$6c. Summed Feature 9 comprised 10-methyl $C_{16:0}$ and/or *iso*-$C_{17:1}$ ω9c.

Table S3 Biosynthetic gene clusters (⩾50% similarity with known bio-clusters) found in antiSMASH ver.7.0 for the *Nonomuraea* strains in this study.

Region	Type	From	To	Most similar known cluster to	MIBiG BGC-ID
***Nonomuraea* sp. FMUSA5-5^T**					
Region 3.1	T1PKS, T3PKS	1	22,452	6-Methylsalicyclic acid (100% of genes show similarity)	BGC0001276 *Aspergillus terreus*
Region 58.1	T1PKS, Phenazine, NRPS	121,567	205,302	Phenazine SA (56% of genes show similarity)	BGC0002561 *Streptomyces* sp.
Region 60.2	Lassopeptide	34,733	57,357	Citrulassin E (80% of genes show similarity)	BGC0001551 *Streptomyces glaucescens*
Region 60.3	Cyanobactin, NRP-metallophore, NRPS	75,998	168,541	Coelichelin (63% of genes show similarity)	BGC0000325 *Streptomyces coelicolor* A3(2)
Region 60.4	Terpene	170,772	191,409	2-Methylisoborneol (100% of genes show similarity)	BGC0000657 *Pseudanabaena* sp. dqh15
Region 72.1	NAPAA	28,974	62,804	ε-Poly-L-lysine (100% of genes show similarity)	BGC0002174 *Epichloe festucae*
Region 72.2	Lanthipeptide-class-iii, Terpene	105,368	148,631	Catenulipeptin (60% of genes show similarity)	BGC0000501 *Catenulispora acidiphila* DSM 44928
Region 90.1	Terpene	71,009	93,252	Geosmin (100% of genes show similarity)	BGC0000661 *Nostoc punctiforme* PCC 73102
***Nonomuraea candida* NRRL B-24552^T**					
Region 3.2	Terpene	303,010	325,274	Geosmin (100% of genes show similarity)	BGC0000661 *Nostoc punctiforme* PCC 73102
Region 42.1	Lassopeptide	20,665	43,230	Achromosin (50% of genes show similarity)	BGC0001493 *Streptomyces achromogenes* subsp. *achromogenes*
Region 53.1	Lanthipeptide-class-iii	33,318	54,809	Catenulipeptin (60% of genes show similarity)	BGC0000501 *Catenulispora acidiphila* DSM 44928
***Nonomuraea aridisoli* KC333^T**					
Region 88.1	Terpene	22,016	44,187	Geosmin (100% of genes show similarity)	BGC0000661 *Nostoc punctiforme* PCC 73102
Region 177.1	Thiopeptide, LAP	21,978	52,870	GE2270 (100% of genes show similarity)	BGC0001155 *Planobispora rosea*
Region 432.1	T2PKS	1	42,980	Persiamycin A (60% of genes show similarity)	BGC0002045 *Streptomonospora* sp. PA3
***Nonomuraea gerenzanensis* L70^T**					
Region 1.1	NRPS, T3PKS	1,553,055	1,636,644	A40926 (89% of genes show similarity)	BGC0000289 *Nonomuraea gerenzanensis*
Region 1.14	Terpene, Thioamitides, LAP	6,534,357	6,569,938	2-Methylisoborneol (100% of genes show similarity)	BGC0000657 *Pseudanabaena* sp. dqh15
Region 1.15	NRP-metallophore, NRPS	6,595,394	6,652,686	Coelichelin (63% of genes show similarity)	BGC0000325 *Streptomyces coelicolor* A3(2)
Region 1.16	NRPS, T1PKS	7,201,618	7,258,831	5-Dimethylallylindole-3-acetonitrile (55% of genes show similarity)	BGC0002128 *Streptomyces coelicolor* A3(2)
Region 1.18	NAPAA	8,317,258	8,351,100	ε-Poly-L-lysine (100% of genes show similarity)	BGC0002174 *Epichloe festucae*
Region 1.20	Lanthipeptide-class-iii	8,426,040	8,448,613	Catenulipeptin (60% of genes show similarity)	BGC0000501 *Catenulispora acidiphila* DSM 44928
Region 1.29	Terpene	11,726,682	11,748,922	Geosmin (100% of genes show similarity)	BGC0000661 *Nostoc punctiforme* PCC 73102

Table S4 Biosynthetic gene clusters (<50% similarity with known bio-clusters) found in antiSMASH Ver.7.0 for the *Nonomuraea* strains in this study.

Region	Type	From	To	Most similar known cluster to	MIBiG BGC-ID
***Nonomuraea* sp. FMUSA5-5[T]**					
Region 1.1	NRPS	168,515	224,887	Salinamide A (21% of genes show similarity)	BGC0001230 *Streptomyces* sp. CNB091
Region 56.1	Terpene	71,880	92,983	-	-
Region 57.1	Terpene	54,226	75,092	-	-
Region 60.1	T1PKS	1	31,538	Maklamicin (8% of genes show similarity)	BGC0001288 *Micromonospora* sp. GMKU326
Region 61.1	Thiopeptide, LAP	96,782	126,310	-	-
Region 61.2	hgIE-KS, Other, Proteusin	347,995	431,525	Depsibosamycin B (19% of genes show similarity)	BGC0002360 *Streptomyces aurantiacus*
Region 65.1	Terpene	100,326	121,366	-	-
Region 66.1	T1PKS	76,909	122,503	Azicemicin B (6% of genes show similarity)	BGC0000202 *Kibdelosporangium* sp. MJ126-NF4
Region 69.1	NI-Siderophore	25,243	38,487	-	-
Region 76.1	T1PKS	60,762	104,571	Everninomicin A (4% of genes show similarity)	BGC0002088 *Micromonospora carbonacea africana*
Region 77.1	Thioamide-NRP, 2dos	1	51,136	Disgocidine (23% of genes show similarity)	BGC0001147 *Streptomyces netropsis*
Region 80.1	Betalactone	14,338	46,898	Frankobactin A1 (12% of genes show similarity)	BGC0002409 *Frankia* sp. CH37
Region 81.1	Nucleoside	25,603	46,322	-	-
Region 84.1	T2PKS	51,520	102,913	Xantholipin (48% of genes show similarity)	BGC0000279 *Streptomyces flavogriseus*
Region 91.1	Lassopeptide	30,226	52,676	-	-
Region 92.1	T1PKS, Ladderane, Arylpolyene, NRPS	1	88,841	Atratumycin (31% of genes show similarity)	BGC0001975 *Streptomyces atratus*
Region 101.1	NI-Siderophore	24,998	38,002	Peucechelin (10% of genes show similarity)	BGC0002466 *Streptomyces peucetius* subsp. *caesius* ATCC 27952
Region 102.1	NRPS	1	52,360	Persiamycin A (5% of genes show similarity)	BGC0002045 *Streptomonospora* sp. PA3
Region 112.1	Other	12,830	53,441	Oryzanaphthopyran A (6% of genes show similarity)	BGC0002656 *Streptacidiphilus oryzae* TH49
Region 120.1	T3PKS	8,279	47,895	Lagunapyrone A (22% of genes show similarity)	BGC0001647 *Streptomyces* sp. MP131-18
Region 127.1	Terpene	206,384	227,499	-	-
Region 138.1	Terpene	122,962	143,963	Chlortetracycline (5% of genes show similarity)	BGC0000209 *Kitasatospora aureofaciens*
Region 146.1	CDPS	1	14,418	Purincyclamide (40% of genes show similarity)	BGC0001986 *Streptomyces chrestomyceticus*
***Nonomuraea candida* NRRL B-24552[T]**					
Region 1.1	Terpene	38,483	59,574	Chlortetracycline (5% of genes show similarity)	BGC0000209 *Kitasatospora aureofaciens*
Region 2.1	Terpene	197,605	218,642	-	-
Region 3.1	Lanthipeptide-class-i	173,654	198,788	-	-
Region 3.3	NI-Siderophore	357,953	371,348	-	-
Region 6.1	Other	40,267	80,704	C-1027 (12% of genes show similarity)	BGC0001584 *Streptomyces* sp. CB02366
Region 6.2	NRPS, T1PKS, NRP-Metallophore, Betalactone	99,454	199,595	Kedarcidin (16% of genes show similarity)	BGC0000081 *Streptoalloteichus* sp. ATCC 53650
Region 8.1	Lassopeptide	235,245	257,840	LP2 (33% of genes show similarity)	BGC0002289 *Streptomyces leeuwenhoekii*
Region 9.1	NRPS	1	51,305	Mannopeptimycin (33% of genes show similarity)	BGC0000388 *Streptomyces hygroscopicus*
Region 14.1	Lanthipeptide-class-ii, LAP, NRPS	4,756	59,902	Goadsporin (12% of genes show similarity)	BGC0000565 *Streptomyces* sp. TP-A0584
Region 14.2	Other	114,928	155,890	Phosphinothricintripeptide (34% of genes show similarity)	BGC0000406 *Streptomyces viridochromogenes*
Region 14.3	T1PKS	162,754	209,259	Efomycin K (12% of genes show similarity)	BGC0002291 *Streptomyces* sp. M56
Region 14.4	T2PKS	221,437	293,904	Xantholipin (16% of genes show similarity)	BGC0000279 *Streptomyces flavogriseus*
Region 14.5	T1PKS	301,460	348,018	Sceliphrolactam (16% of genes show similarity)	BGC0001770 *Streptomyces* sp. SD85
Region 14.6	Arylpolyene, T1PKS, NRPS	379,306	434,985	Amipurimycin (16% of genes show similarity)	BGC0001957 *Streptomyces novoguineensis*
Region 16.1	T1PKS	17,882	63,506	Murayaquinone (5% of genes show similarity)	BGC0001675 *Streptomyces griseoruber*
Region 27.1	Thioamitides	24,650	47,303	-	-
Region 27.2	Lassopeptide	58,718	81,038	Canucin A (28% of genes show similarity)	BGC0002318 *Streptomyces canus*
Region 28.1	Lassopeptide	120,092	142,699	LP2 (33% of genes show similarity)	BGC0002289 *Streptomyces leeuwenhoekii*
Region 31.1	T3PKS	1	40,394	Lagunapyrone A (22% of genes show similarity)	BGC0001647 *Streptomyces* sp. MP131-18
Region 34.1	NI-Siderophore	23,604	36,759	Peucechelin (15% of genes show similarity)	BGC0002466 *Streptomyces peucetius* subsp. *caesius* ATCC 27952
Region 41.1	Lanthipeptide-class-ii	36,213	59,254	Trichostatin RK (13% of genes show similarity)	BGC0002364 *Streptomyces* sp.
Region 43.1	Lanthipeptide-class-i	5,070	30,245	-	-

Table S4 Biosynthetic gene clusters (<50% similarity with known bio-clusters) found in antiSMASH Ver.7.0 for the *Nonomuraea* strains in this study. (*Cont.*)

**Region**	**Type**	**From**	**To**	**Most similar known cluster to**	**MIBiG BGC-ID**
Region 44.1	NRPS	1	47,486	Paulomycin (3% of genes show similarity)	BGC0001732 *Streptomyces* sp. YN86
Region 47.1	Betalactone	33,342	65,361	Frankobactin A1 (12% of genes show similarity)	BGC0002409 *Frankia* sp. CH37
Region 48.2	Ranthipeptide	74,392	96,193	-	-
Region 48.1	NRPS, T1PKS	14,631	65,015	Collismycin A (11% of genes show similarity)	BGC0000973 *Streptomyces* sp. CS40
Region 59.1	Lanthipeptide-class-iv	69,132	87,460	Blasticidin S (7% of genes show similarity)	BGC0000874 *Streptomyces griseochromogenes*
Region 63.1	T1PKS, Lipolanthine, Lanthipeptide-class-iii	1	33,389	Guadinomine (7% of genes show similarity)	BGC0000998 *Streptomyces* sp. K01-0509
Nonomuraea aridisoli **KC333^T**					
Region 34.1	Terpene	1	16,090	-	-
Region 53.1	T3PKS	1	21,761	Lagunapyrone A (22% of genes show similarity)	BGC0001647 *Streptomyces* sp. MP131-18
Region 102.1	NI-Siderophore	7,870	17,985	-	-
Region 112.1	Terpene	1	17,448	Chlortetracycline (5% of genes show similarity)	BGC0000209 *Kitasatospora aureofaciens*
Region 122.1	T1PKS	34,833	61,867	-	-
Region 131.1	Terpene	555	16,346	Frankiamicin (14% of genes show similarity)	BGC0001197 *Frankia* sp. EAN1pec
Region 158.1	NI-Siderophore	1	12,366	Nonactin (33% of genes show similarity)	BGC0000244 *Streptomyces griseus* subsp. *griseus*
Region 167.1	NRPS	1	13,796	-	-
Region 344.1	NRPS	21,994	45,624	Quinolidomicin A (5% of genes show similarity)	BGC0002520 *Micromonospora* sp.
Region 355.1	Lanthipeptide-class-i	132	26,528	Dechlorocuracomycin (8% of genes show similarity)	BGC0001569 *Streptomyces noursei* ATCC 11455
Region 543.1	Lanthipeptide-class-i	1	17,138	-	-
Region 568.1	NI-Siderophore	15,114	28,164	Peucechelin (15% of genes show similarity)	BGC0002466 *Streptomyces peucetius* subsp. *caesius* ATCC 27952
Nonomuraea gerenzanensis **L70^T**					
Region 1.2	Terpene	1,869,659	1,889,297	-	-
Region 1.3	Lanthipeptide-class-i	3,342,482	3,366,327	-	-
Region 1.4	Terpene	3,470,071	3,489,732	Chlortetracycline (5% of genes show similarity)	BGC0000209 *Kitasatospora aureofaciens*
Region 1.5	NRPS	4,136,205	4,190,363	Cinnapeptin (10% of genes show similarity)	BGC0002108 *Streptomyces viridosporus* ATCC 14672
Region 1.6	T1PKS, Butyrolactone	4,380,740	4,428,779	-	-
Region 1.7	Terpene	4,853,531	4,873,825	-	-
Region 1.8	Ectoine	5,141,174	5,151,548	Kosinostatin (6% of genes show similarity)	BGC0001073 *Micromonospora* sp. TP-A0468
Region 1.9	T1PKS	5,185,357	5,229,523	C-1027 (32% of genes show similarity)	BGC0001584 *Streptomyces* sp. CB02366
Region 1.10	Lanthipeptide-class-I, hgIE-KS	5,390,485	5,443,363	Ibomycin (4% of genes show similarity)	BGC0001619 *Streptomyces* sp. WAC2288
Region 1.11	Terpene, NRPS	5,704,605	5,760,050	Isorenieratene (25% of genes show similarity)	BGC0001456 *Streptomyces argillaceus*
Region 1.12	T1PKS	6,221,232	6,258,670	MS-271 (14% of genes show similarity)	BGC0001674 *Streptomyces mirabilis*
Region 1.13	T1PKS	6,432,366	6,475,313	Everninomicin A (4% of genes show similarity)	BGC0002088 *Micromonospora carbonacea africana*
Region 1.17	Betalactone	8,153,088	8,185,843	Frankobactin A1 (12% of genes show similarity)	BGC0002409 *Frankia* sp. CH37
Region 1.19	Lanthipeptide-class-ii	8,395,784	8,418,858	-	-
Region 1.21	Terpene	8,484,625	8,505,174	Frankiamicin (14% of genes show similarity)	BGC0001197 *Frankia* sp. EAN1pec
Region 1.22	Terpene	9,011,287	9,030,974	-	-
Region 1.23	T2PKS	9,126,867	9,199,346	Pradimicin-A (28% of genes show similarity)	BGC0000256 *Actinomadura hibisca*
Region 1.24	T3PKS	9,282,876	9,323,895	Lagunapyrone A (22% of genes show similarity)	BGC0001647 *Streptomyces* sp. MP131-18
Region 1.25	Blactam	9,714,267	9,736,141	Tabtoxin (13% of genes show similarity)	BGC0000846 *Pseudomonas syringae*
Region 1.26	NI-Siderophore	10,557,681	10,568,515	Peucechelin (15% of genes show similarity)	BGC0002466 *Streptomyces peucetius* subsp. *caesius* ATCC 27952
Region 1.27	T1PKS	11,187,647	11,233,148	Murayaquinone (5% of genes show similarity)	BGC0001675 *Streptomyces griseoruber*
Region 1.28	NI-Siderophore	11,685,339	11,698,631	-	-
Region 2.1	Lanthipeptide-class-v	1	37,595	Pristinin A3 (11% of genes show similarity)	BGC0002303 *Streptomyces pristinaespiralis*

Table S5. General features of the genome sequences of strain FMUSA5-5^T and its closely related type strains**.**

Features	Strain FMUSA5-5^T	*Nonomuraea candida* NRRL B-24552^T	*Nonomuraea aridisoli* KC333^T	*Nonomuraea gerenzanensis* L70^T
**Bioproject**	PRJNA613987	PRJNA238534	PRJNA429095	PRJNA765517
**Accession no.**	JAATEP000000000	JOAG00000000	POUD00000000	CP084058
**Genome coverage**	51x	136x	30x	87x
**N50**	187,133	217,444	27,064	12,010,032
**Number of Contigs**	187	240	729	3
**Genome size (Mbp)**	12.4	11.0	9.9	12.1
**DNA G+C content (%)**	71.5	72.0	71.0	71.5
**Number of genes**	11,583	10,226	9,771	11,279
**Protein coding genes**	11,142	9,884	9,279	10,956
**Number of RNAs**	68	83	85	91
**rRNA**	2	15	17	15
**tRNA**	63	65	65	73
**Other RNA**	3	3	3	3
**Pseudogene**	373	259	407	232

Table S6. ANI, AAI, and Digital DNA-DNA hybridization relatedness values of strain FMUSA5-5^T and the closest type strains.

Query genome	Reference genome	ANIb (%)	AAI (%)	Digital DNA-DNA hybridization relatedness Formula 2*				G+C difference
				% dDDH	Model C.I. (%)	Distance	Prob. DDH ≥ 70 (same species)	
Strain FMUSA5-5^T	*Nonomuraea candida* NRRL B-24552^T	85.8	83.2	33.8	31.4 – 36.3	0.1235	0.44	0.5
	Nonomuraea aridisoli KC333^T	82.8	79.4	29.5	27.2 – 32.0	0.1447	0.09	0.5
	Nonomuraea gerenzanensis L70^T	88.3	87.3	38.5	36.0 – 41.0	0.1046	1.84	0.0

*Formula 2: a formula (identities/HSP length) that is liberated of genome length and is thus prosperous against the use of incomplete draft genomes.

Table S7. Biological activity of compound **1** (Pradimicin U).

**Compounds**	**Anti-*P. falciparum*[a] (K1 strain) (IC_{50}, μg/mL)**	**Anti-*M. tuberculosis*[b] (H37Ra strain) (MIC, μg/mL)**	**Anti-*A. brassicicola*[b] (MIC, μg/mL)**	**Anti- *B. cereus*[b] (MIC, μg/mL)**	**Anti- *S. aureus*[b] (MIC, μg/mL)**	**Anti- *A. baumannii*[b] (MIC, μg/mL)**	**DPPH radical scavenging activity[c] IC_{50} (μg/mL)**	**Cytotoxicity against NCI-H187 cell (IC_{50}, μg/mL)[b]**	**Cytotoxicity against MCF-7 cell (IC_{50}, μg/mL)[b]**	**Cytotoxicity against Vero cell (IC_{50}, μg/mL)[b]**
Compound **1** (Pradimicin U)	3.65	25.0	25.0	6.25	1.56	Inactive	52.1	5.69	52.49	21.84
Dihydroartemisinin	0.0025									
Chloroquine	0.129									
Streptomycin		1.25								
Isoniazid		0.09								
Ofloxacin		0.78								
Ethambutol		0.94								
Amphotericin B			0.781							
Rifampicin		0.013		0.625	0.0781	3.13				
Vancomycin				4.1	1.05					
Erythromycin						25.0				
BHT							69.4			
Doxorubicin								0.056	8.64	
Tamoxifen									8.06	
Ellipticine								3.62		0.72

[a] Maximum tested concentration was done at 10 μg/mL.
[b] Maximum tested concentration was done at 100 μg/mL.

[c] Maximum tested concentration was done at 1000 μg/mL.

TBRC culture collection certificate

BIOTEC
a member of NSTDA

Thailand Bioresource Research Center (TBRC)
National Center for Genetic Engineering and Biotechnology
Innovation Cluster 2 (Tower B, 8th Floor, Room 817)
143 Thailand Science Park, Phahonyothin Road
Khlong Nueng, Khlong Luang, Pathum Thani 12120, Thailand
Tel +66-2-1178000-1, Fax: +66-2-1178003

ใบรับรองการฝาก
CERTIFICATE OF DEPOSIT

ใบรับรองเลขที่ / Certificate number: 2018-028

วันที่ออกใบรับรอง / Date of issue: 20-Jun-2018

ชื่อผู้ฝาก / Depositor: Assoc. Prof. Dr. Chitti Thawai

หน่วยงานและที่อยู่ / Institute and address: Department of Biology, Faculty of Science, King Mongkut's Institute of Technology Ladkrabang Bangkok 10520, Thailand

ขอรับรองว่าชีววัสดุที่มีรายชื่อต่อท้ายนี้ได้นำมาฝากเก็บรักษาและดูแล ณ Thailand Bioresource Research Center (TBRC) และได้ให้เลขที่รหัสประจำตัวชีววัสดุไว้เป็นหลักฐานการรับฝาก

This is to certify that the following biological material(s) has/have been maintained at Thailand Bioresource Research Center (TBRC). Accession number(s) of the biological material(s) is/are the evidence of deposition.

รายชื่อชีววัสดุ / List of biological materials

ชื่อชีววัสดุ<br>Biological material name	รหัสชีววัสดุ<br>Accession number	ระยะเวลาการฝาก<br>Deposit period	หมายเหตุ*<br>Remark
Micromonospora sp. HM5-17	TBRC 8477	Since 3 April 2018	Remark 2
Micromonospora sp. PPF5-17	TBRC 8478	Since 3 April 2018	Remark 2
Micromonospora sp. PPF5-6	TBRC 8479	Since 3 April 2018	Remark 2
Nonomuraea sp. FMUSA5-5	TBRC 8481	Since 3 April 2018	Remark 2
Actinomadura sp. AT2L-12	TBRC 8482	Since 3 April 2018	Remark 2
Streptomyces sp. AA8	TBRC 8483	Since 3 April 2018	Remark 2
Mumia sp. BB11-22	TBRC 8484	Since 3 April 2018	Remark 2

* Remark 1 = TBRC จัดตัวอย่างดังกล่าวอยู่ในส่วนที่เผยแพร่แก่สาธารณะได้ ข้อมูลตัวอย่างจะเผยแพร่ในแค็ตตาล็อกหลังจาก TBRC ออกใบรับรองการฝาก
The biological material is available in the publicly accessible section of the TBRC. The biological material will be included in the online catalog after TBRC issues a certificate of deposit.

Remark 2 = TBRC จัดตัวอย่างดังกล่าวอยู่ในส่วนที่เผยแพร่แก่สาธารณะได้ และไม่ได้วางข้อกำหนดในการเข้าถึงข้อมูลเรื่องการมีอยู่ของตัวอย่างดังกล่าว ณ TBRC ข้อมูลตัวอย่างจะเผยแพร่ในแค็ตตาล็อกหลังจากได้รับการตีพิมพ์
The biological material is available in the publicly accessible section of the TBRC and restrictions have not been placed on access to information concerning the presence of the biological material in the TBRC. The biological material will be included in the online catalog after publication.

Remark 3 = ไม่เผยแพร่แก่สาธารณะ
The material is not available for public access.

ลายเซ็น / Signature Chanwit Suriyachadkun

ผู้รับฝาก / Curator Dr. Chanwit Suriyachadkun

NBRC culture collection certificate

NITE Biological Resource Center

(1/2)

生物遺伝資源受託証
Notice of Acceptance of Biological Material

平成３０年８月２７日
Date of Issue: August 27, 2018

寄託者 / Depositor
Chanwit Suriyachadkun
BIOTEC Culture Collection, National Center for Genetic Engineering and Biotechnology (BIOTEC)

独立行政法人製品評価技術基盤機構
バイオテクノロジーセンター所長　木井　保夫
Yasuo Kii
Director-General
Biological Resource Center
National Institute of Technology and Evaluation（NITE）

このたび貴殿よりお預かりしました生物遺伝資源につきましては、下記のとおり NBRC 番号を付与し、寄託を受けましたのでお知らせいたします。NITE バイオテクノロジーセンターは、寄託を受け入れた生物遺伝資源を一般に公開し、非商業的使用に対して分譲します。
This is to notify you that the following biological material(s) received from you has (have) been accepted with the following NBRC accession number(s).When requested, Biological Resource Center, NITE (NBRC) will distribute the deposited biological material(s) to anyone including company for non-commercial use.

記
NOTICE

Name of biological material(s) (Strain ID)	NBRC number	Date of acceptance
(1) *Micromonospora* sp. (TBRC 8478)	NBRC 113441	July 11, 2018
(2) *Micromonospora* sp. (TBRC 8479)	NBRC 113442	July 11, 2018
(3) *Nonomuraea* sp. (TBRC 8481)	NBRC 113443	July 11, 2018
(4) *Actinomadura* sp. (TBRC 8482)	NBRC 113444	July 11, 2018
(5) *Mumia* sp. (TBRC 8484)	NBRC 113445	July 11, 2018

※ ISO9001登録マークは、製品またはサービスそのものを保証するものではありません。
ISO9001 registration mark does not guarantee the quality of products or services themselves.

JQA-QMA13036
ＮＢＲＣ

National Institute of Technology and Evaluation
独立行政法人 製品評価技術基盤機構

(6) *Gordonia* sp. (TBRC 8485)	NBRC 113446	July 11, 2018
(7) *Quadrisphaera* sp. (TBRC 8486)	NBRC 113447	July 11, 2018
(8) *Nonomuraea* sp. (TBRC 8487)	NBRC 113448	July 11, 2018
(9) *Amycolatopsis* sp. (TBRC 8488)	NBRC 113449	July 11, 2018
(10) *Microbispora* sp. (TBRC 8489)	NBRC 113450	July 11, 2018
(11) *Streptomyces* sp. (TBRC 8483)	NBRC 113461	July 18, 2018
